# Baby-OSCAR: Outcome after Selective early treatment for Closure of patent ductus ARteriosus in preterm babies—a statistical analysis plan for short-term outcomes

**DOI:** 10.1186/s13063-021-05324-3

**Published:** 2021-05-26

**Authors:** Jennifer L. Bell, Samir Gupta, Edmund Juszczak, Pollyanna Hardy, Louise Linsell

**Affiliations:** 1 University of Oxford, Oxford, UK; 2 Durham University, Durham, UK; 3 University of Nottingham, Nottingham, UK; 4 University of Birmingham, Birmingham, UK

**Keywords:** Newborn, Patent ductus arteriosus, PDA, Echocardiography, Preterm, Ibuprofen, Bronchopulmonary dysplasia, Statistical analysis plan, Randomised controlled trial

## Abstract

**Background:**

The Baby-OSCAR trial is a multi-centre, randomised, placebo-controlled parallel group trial of early treatment of large patent ductus arteriosus (PDA) with ibuprofen in extremely preterm infants. This paper describes the statistical analysis plan for the short-term health outcomes of the Baby-OSCAR trial.

**Methods and design:**

This is a randomised controlled trial to determine if early-targeted treatment of a large PDA with parenteral ibuprofen in extremely preterm babies improves short and long-term health and economic outcomes. Infants born between 23^+0^ and 28^+6^ weeks of gestation, confirmed by echocardiography as having a large PDA (with a diameter of at least 1.5 mm and unrestricted pulsatile PDA flow pattern), with parental informed consent, were randomly allocated to receive either ibuprofen or placebo within 72 h of birth. The primary outcome is a composite of death by 36 weeks’ postmenstrual age or moderate or severe bronchopulmonary dysplasia (BPD) at 36 weeks’ postmenstrual age.

**Results:**

Baseline demographic and clinical characteristics will be described by randomised group. The primary analysis will be on the modified intention to treat (ITT) population. Counts and percentages will be presented for binary and categorical variables, and mean and standard deviation or median and interquartile range will be presented for continuous variables. For binary outcomes, risk ratios and confidence intervals will be calculated using log binomial or Poisson regression with a robust variance estimator. Continuous outcomes will be analysed using linear regression models, or quantile regression models if skewed. Analyses will be adjusted for all minimisation factors where technically possible, and correlation between siblings from multiple births will be accounted for by nesting the clusters as a random effect. Both crude and adjusted effect estimates will be presented, with the primary inference based on the adjusted estimates. Ninety-five per cent confidence intervals will be used for all pre-specified outcome comparisons.

**Conclusion:**

This paper describes the statistical analysis plan for short-term health outcomes of the trial, including the analysis principles, definitions of important outcomes, methods for primary analysis, pre-specified subgroup analysis, and secondary analysis. The plan was finalised prior to completion of short-term follow-up.

**Trial registration:**

ISRCTN registry ISRCTN84264977. Registered on 15 September 2010.

**Supplementary Information:**

The online version contains supplementary material available at 10.1186/s13063-021-05324-3.

## Introduction

This paper details the proposed presentation and analyses for the main paper(s) reporting results from the National Institute for Health Research (NIHR) Health Technology Assessment (HTA) programme funded multicentre randomised controlled trial of early treatment for closure of large Patent Ductus Arteriosus (PDA) with ibuprofen in extremely preterm infants (Baby-OSCAR).

Baby-OSCAR is a randomised controlled trial to determine short- and long-term health and economic outcomes of the treatment of a large PDA in extremely preterm babies with ibuprofen within 72 h of birth. Infants who were born between 23^+0^ and 28^+6^ weeks of gestation, confirmed by echocardiography as having a large PDA, were randomised to receive either ibuprofen or placebo within 72 h of birth. The primary outcome is a composite of death by 36 weeks of postmenstrual age, or moderate or severe bronchopulmonary dysplasia (BPD) at 36 weeks of postmenstrual age [1].

This paper describes the statistical analysis plan for the short-term outcomes of the main trial, including the analysis principles, definitions of outcomes, methods for primary analysis, pre-specified subgroup analysis, and secondary analysis. The analyses for both the long-term outcomes at 2 years of age and the health economic evaluation will be detailed in separate publicly-available analysis plans.

This statistical analysis plan conforms to the published guidelines on the content for statistical analysis plans in clinical trials [2] and was finalised prior to completion of short-term follow-up to the trial. Any deviations from this plan will be described and justified in the final report of the trial.

## Background information

### Rationale

This trial follows an internal pilot phase, which has been run to assess the suitability of trial procedures and likelihood of recruitment targets being achieved. The trial aimed to recruit approximately 730 infants in total (including those recruited during the internal pilot phase) from 35 UK tertiary neonatal units (and five for the internal pilot phase).

PDA is associated with a number of serious and life-threatening short and long term complications including low blood pressure (hypotension), bleeding in the lungs (pulmonary haemorrhage) and brain (intraventricular haemorrhage (IVH)), systemic complications such as necrotising enterocolitis (NEC), bronchopulmonary dysplasia (BPD), and long term health problems such as neurodevelopmental disability and chronic respiratory problems. The persistence of PDA is associated with an 8-fold rise in neonatal mortality [3]. In addition, as PDA is very common in extreme preterm babies and is associated with a prolonged need for respiratory support and hospitalisation, it places a significant financial burden on the National Health Service (NHS).

Historically, clinicians who have been concerned about the complications associated with a PDA have attempted to close PDAs utilising medical (pharmacological) or surgical treatment. Traditionally, medical treatment is instituted as prophylactic treatment (within 24 h of birth) or symptomatic treatment (usually 5−7 days after birth). Prophylactic pharmacological treatment of all preterm babies unnecessarily exposes a large proportion of babies to the potentially serious side effects of drug treatment, when their PDA would have closed spontaneously. Symptomatic treatment on the contrary delays treatment whilst waiting for symptoms to appear and could result in a loss of treatment benefit as irreversible damage may have already been done.

Moreover, the practice of a conservative approach of not treating seems to originate from uncertainty regarding the management of PDA rather than evidence favouring no intervention. This is because most studies conducted to date have involved more mature preterm babies (over 1000 g or 28 weeks of gestation) whose PDA is more likely to close spontaneously. The studies were also largely designed to assess PDA closure rates rather than clinically important outcomes.

It is now suggested that large PDAs (those with a diameter of ≥ 1.5 mm) through which blood flow is pulsatile and unrestricted are less likely to close spontaneously. Targeted early treatment of large PDAs whilst asymptomatic has the potential to overcome the disadvantages of both the prophylactic and symptomatic approaches. Although clinical detection of PDA whilst asymptomatic is challenging, it can be assessed using bedside echocardiography.

Non-steroidal anti-inflammatory drugs, especially indomethacin and ibuprofen have been widely used for the treatment of PDA. Short-term efficacy of indomethacin and ibuprofen are equivalent in the treatment of PDA [4]. Ibuprofen however appears to reduce the risk of NEC and is associated with fewer clinical gastrointestinal and renal side effects compared to indomethacin; hence it is the drug of choice for this trial. Paracetamol has also been recently reported in case studies for closure of symptomatic PDA but further research needs to be done to establish its effectiveness [5].

The aim of this trial is to examine whether the pharmacological closure of a large PDA (identified by echocardiography) in extremely preterm babies whilst asymptomatic has a clinically important impact on both short- and long-term health and economic outcomes.

### Objectives of the trial

The primary objective of the trial is to determine if selective early treatment of large PDAs (confirmed by echocardiograph) in extremely preterm babies with ibuprofen within 72 h of birth reduces the incidence of death by 36 weeks of postmenstrual age or moderate or severe bronchopulmonary dysplasia (BPD) at 36 weeks of postmenstrual age.

The secondary objectives are to determine if the selective treatment of confirmed large PDAs in extremely preterm babies with ibuprofen within 72 h of birth results in:

- A reduction in the components of the primary outcome: death by 36 weeks of postmenstrual age; moderate or severe BPD at 36 weeks of postmenstrual age, severity of BPD at 36 weeks of postmenstrual age; other secondary outcomes up to discharge (see the ‘Secondary short-term outcomes’ section);
- Improved health outcomes at 2 years’ corrected age including survival without moderate or severe neurodevelopmental disability (long-term primary objective) and survival without respiratory morbidity (long-term secondary objective).

An economic evaluation will be carried out from the perspective of the health service. It will take the form of a cost-effectiveness analysis presented in terms of cost per major outcome averted. The major outcomes are those of the primary outcome, namely death and moderate or severe BPD by 36 weeks of postmenstrual age. Additional analyses will take place on a range of secondary outcomes and on neurodevelopmental outcomes at 2 years. The incremental cost estimate for statistically significant differences in the pre-specified outcomes in primary and subgroup analyses would be computed.

### Trial design

This is a multicentre, randomised, placebo-controlled parallel group trial to determine if the treatment of a large PDA with ibuprofen in extremely preterm babies (23^+0^ to 28^+6^ weeks of gestation) improves short- and long-term health outcomes and health economic outcomes.

The main trial was preceded by an internal pilot phase, which was used to assess the suitability of trial procedures and likelihood of recruitment targets being achieved.

The entire trial is anticipated to take 82 months to complete and aims to recruit a total of approximately 730 extremely preterm babies.

### Eligibility

#### Inclusion criteria

Babies will be considered eligible for inclusion into the trial if they are:

- Born at 23^+0^ to 28^+6^ weeks of gestation
- Less than 72 h old
- Confirmed by echocardiography as having a large PDA which
  - Is at least 1.5 mm in diameter (determined by gain optimised colour Doppler), and
  - Has unrestrictive pulsatile (left to right) flow (ratio of flow velocity in PDA Maximum (V_max_) to Minimum (V_min_) > 2:1) or, growing flow pattern (< 30% right to left), and no clinical concerns of pulmonary hypertension.

In addition:

- The responsible clinician is uncertain about whether the baby might benefit from treatment to close the PDA
- Written informed consent has been obtained from the parent(s)

#### Exclusion criteria

Babies will be excluded from participation in the trial if they have:

- No realistic prospect of survival
- Severe congenital anomaly
- Clinical or echocardiography suspicion of congenital structural heart disease that contraindicates treatment with ibuprofen
- Other conditions that would contraindicate the use of ibuprofen (active bleeding especially intracranial or gastrointestinal bleeding, coagulopathy, thrombocytopenia (platelet count < 50,000), renal failure, life-threatening infection, pulmonary hypertension, known or suspected necrotising enterocolitis (NEC))
- Indomethacin, ibuprofen, or paracetamol administration after birth

### Interventions

Ibuprofen will be supplied as a clear sterile solution at a concentration of 5 mg/ml in ampoules. Cartons containing four 2-ml single-use ampoules will be provided. Each carton will be labelled with a unique code and in compliance with the guidance given in Annexe 13 of the European Commission’s guidelines for Good Manufacturing Practice.

An initial loading dose of 10 mg/kg (2 ml/kg) of ibuprofen will be administered, followed by two 5-mg/kg (1 ml/kg) doses at 24 and 48 h after the initial dose. Doses are to be calculated on the birth weight of the baby. If required, the IMP can be diluted to the appropriate volume with 5% glucose or 0.9% sodium chloride. Each dose is to be given as a short intravenous infusion over 15 min. All 3 doses will be given unless there are adverse effects necessitating stoppage, as referenced in the trial protocol [1].

Placebo will be supplied as a clear sterile solution of 0.9% sodium chloride for injection. Cartons identical to those for ibuprofen, each containing four identical single-use ampoules will be provided. Volume of IMP to be withdrawn from the ampoule will be calculated following the calculations for ibuprofen dosing.

Following enrolment, the first dose should be administered soon after randomisation, after 6 h of age, and within 72 h of birth. The recommended storage will be in line with the Summary of Product Characteristics (SmPC) and once the ampoule is opened the drug must be used immediately.

### Definition of primary and secondary outcomes

#### Primary outcome

The primary outcome is defined as a composite outcome of death by 36 weeks of postmenstrual age or moderate or severe BPD at 36 weeks of postmenstrual age (as defined in Table 1).

**Table 1:** Severity-based diagnostic criteria for BPD

Time point of assessment:	36 weeks of postmenstrual age
Therapy with oxygen > 21% and/or respiratory support for ≥ 28 days and the following:	
Mild BPD	Baby is breathing room air
Moderate BPD	Baby is in 22–29% oxygen or 0.01–1.0 L/min
Severe BPD	FiO_2_ ≥ 0.3, or low flow oxygen ≥ 1.1 L/min, or the baby is receiving any respiratory support (ventilation, CPAP, or high flow oxygen therapy) to achieve saturations of ≥ 91%

**Fig. 1:**
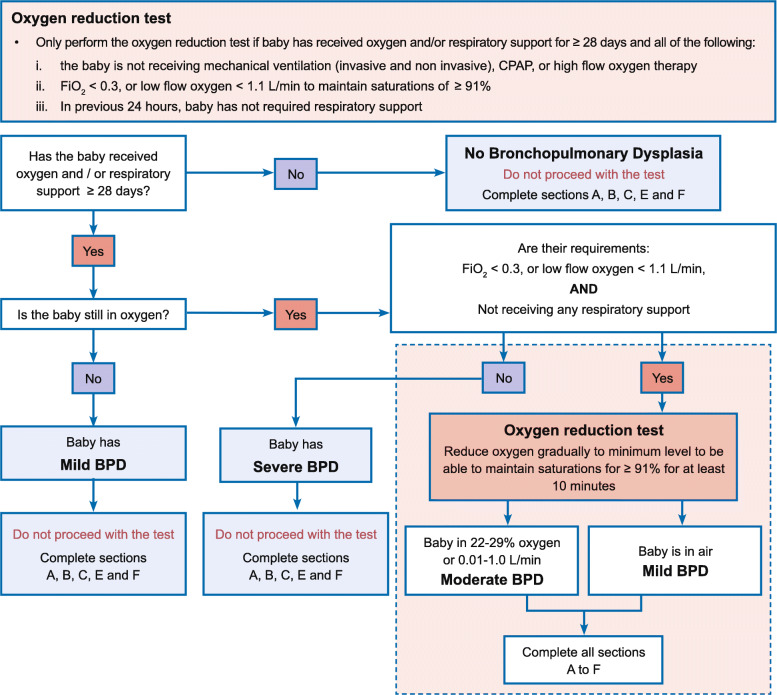
Oxygen reduction test flow chart

The need for oxygen is subjective and hence oxygen dependency to differentiate mild from moderate BPD will be confirmed using an ‘oxygen reduction test’. This is based on the threshold at which the baby is able to maintain oxygen saturations ≥ 91% whilst breathing in air or at a given minimum FiO_2_. Babies unable to maintain oxygen saturations ≥ 91% in room air will be considered to be oxygen dependent and classed as moderate BPD (oxygen requirements 22–29% or 0.01–1.0 L/min by low flow nasal cannula). Those babies who can be weaned to room air and stable will be categorised as having mild BPD. This test will only apply to those babies whose oxygen requirements are < 0.3 or low flow oxygen < 1.1 L/min, and who have not received any additional respiratory support in the previous 24 h. Babies outside of this will not be tested, but their oxygen requirements will be captured on the relevant case report form. See Fig. 1 for the oxygen reduction test flow chart.

#### Secondary short-term outcomes

Due to the multiple number of short-term outcomes and correlation between some outcomes, statistical inference will be restricted to a predefined list of outcomes (see below). Summary statistics by trial arm will be provided for all other outcomes as indicated below, but statistical inferences (i.e. the calculation of confidence intervals) will not be made.

##### Secondary short-term outcomes—statistical inference provided

- Death by 36 weeks’ postmenstrual age
- Moderate or severe BPD at 36 weeks’ postmenstrual age

Incidence or duration of the following up to discharge:

- Severe intraventricular haemorrhage (IVH) (grade III/IV with ventricular dilatation or intraparenchymal abnormality)
- Cystic periventricular leukomalacia (PVL)
- Babies treated for retinopathy of prematurity (ROP)
- Significant pulmonary haemorrhage (fresh blood in endotracheal tube with increase in respiratory support)
- Treated for pulmonary hypertension with pulmonary vasodilator
- NEC definitive and/or complicated (Bell stage II and above) confirmed by radiology and/or histopathology
- Closed or non-significant PDA (< 1.5 mm) at around 3 weeks of age (range of 18–24 days), confirmed by ECHO (or death, or hospital discharge from recruiting centre, if discharged sooner)
- PDA ≥ 1.5 mm at around 3 weeks’ (range of 18–24 days), not treated medically or by surgical closure
- Open-label treatment of a symptomatic PDA by surgical treatment
- Discharge home on oxygen
- Weight gain: a change in z score between birth and discharge (or death if sooner)

##### Secondary short-term outcomes—summarised descriptively

- Severity of BPD at 36 weeks’ postmenstrual age (see Table 1 and Fig. 1).
- Non-cystic PVL
- Hydrocephalus
- NEC requiring surgery
- Gastrointestinal bleeding (leading to investigation or clinical treatment) within 7 days of the first dose of trial drug administration
- Spontaneous intestinal perforation
- Medical open-label treatment of a symptomatic PDA with a COX inhibitor
- Administration and duration of inotropic support
- Total duration of respiratory support
- Invasive ventilation through an endotracheal tube
- Non-invasive support through nasal CPAP, nasal ventilation, humidified high flow nasal cannula therapy, or low flow oxygen ≥ 1.1 L/min
- Duration of initial hospitalisation (birth to discharge home)
- Postnatal steroid use for chronic lung disease
- Tolerance of ibuprofen treatment within the foreseeable SAE reporting range, described in the protocol, section 9.1.4
- Head circumference: a change in head size z score between randomisation and discharge (or death if sooner).

#### Process outcomes

Process outcomes will measure adherence to the protocol – see the ‘Protocol non-compliance’ section below for details.

### Hypothesis framework

This is a superiority trial and all comparisons will be analysed and presented on this basis.

### Sample size and power

Evidence from the TIPP trial suggests that the risk of death or BPD in extremely low birth weight babies at 36 weeks of postmenstrual age allocated placebo is 52% (95% confidence interval [CI] 48% to 56%) [6]. However, this trial investigated the effect of prophylactic treatment and included all babies weighing 500−999 g. More recent information using data derived from the latest report of Neonatal Survey Database from the Trent region [7] provides an approximate rate of death or BPD by 36 weeks of postmenstrual age of 53% for all babies admitted to the neonatal unit. These babies would have been treated according to clinical judgement and therefore a proportion of them would have been treated with ibuprofen. Given that the risk of death or BPD in babies with a large PDA is inherently higher, it is estimated that the risk in this group is 60%.

Su et al [4] compared ibuprofen to indomethacin in babies of ≤ 28 weeks of gestation having a PDA who were less than 24 h old. The combined outcome of death within 30 days or BPD at 36 weeks of postmenstrual age was observed to be 42% (95% CI 29% to 55%).

It is therefore expected, given that babies will be enrolled up to 72 h after birth, that the treatment group incidence of death/BPD at 36 weeks of postmenstrual age will be approximately 48% in the intervention arm. This would imply an absolute risk reduction of 12% (60% to 48%) in the primary outcome of the trial for babies randomised to treatment compared to placebo, which is considered a clinically important difference.

Some babies will require open-label treatment in either the treatment or placebo arm. As open-label treatment should be limited to symptomatic babies meeting only defined criteria, it is considered to have minimal or no effect on the primary outcome. Thus, adjustment of the sample size for open-label treatment is not considered necessary.

**Fig. 2:**
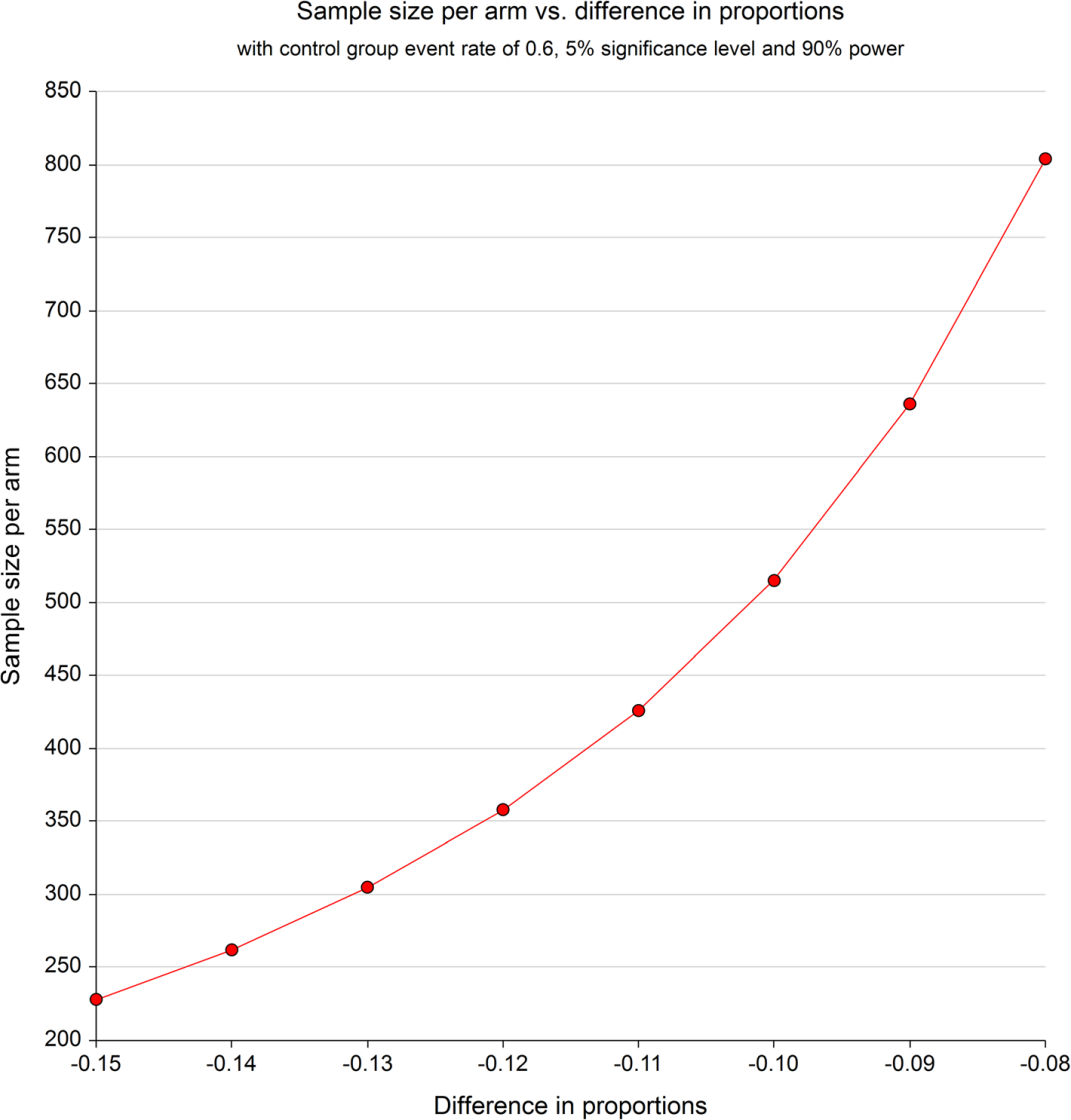
Sample size per arm

Figure 2 depicts a sample size curve for the primary outcome of the trial of death or BPD by 36 weeks of postmenstrual age, assuming 90% power, a two-sided 5% significance level and a 60% control group event rate for the primary outcome.

Table 2 summarises this information and allows for 1% loss to follow-up in the primary outcome. Minimal loss to follow-up is expected for the primary outcome since it is a short-term outcome and recorded whilst the baby is in hospital.

**Table 2:** Sample size required by event rates

Control group event rate	Active Rx group event rate	Absolute risk reduction	Relative risk reduction	Approximate total sample size required
60%	47%	13%	22%	620
**60%**	**48%**	**12%**	**20%**	**730**
60%	49%	11%	18%	870

Thus a sample size of approximately 730 babies in total (365 per arm) would be required to detect an absolute risk reduction of 12% (power 90%, 2-sided significance level of 5%) from a control group event rate of 60% to a treatment group event rate of 48%, assuming 1% lost to follow-up.

n multiple births, the babies are genetically either identical or very similar, so their outcomes are likely to be correlated. However, since multiples will be randomised independently, the loss of precision from multiples randomised to the same arm will be offset by the gain in precision from multiples randomised to the opposite arm, hence there will be a negligible impact on power.

### Intervention allocation

Treatment allocation of ibuprofen or placebo will be in a ratio of 1:1 and masked such that the allocation will not be known by clinicians, the baby’s family or the trial outcome assessors.

Randomisation will be managed via a secure web-based randomisation facility hosted by the NPEU Clinical Trials Unit (CTU) with telephone back-up available at all times (24/7, 365 days a year). The randomisation programme will use a minimisation algorithm to ensure balance between the groups with respect to the size of the PDA (1.5 mm to < 2.0 mm; 2.0 mm to < 3.0 mm; ≥ 3.0 mm), gestational age at birth (23 to 23^+6^ weeks; 24 to 24^+6^ weeks; 25 to 25^+6^ weeks; 26 to 26^+6^ weeks; 27 to 27^+6^ weeks; 28 to 28^+6^ weeks), age at randomisation (< 12 h; 12 to < 24 h; 24 to < 48 h; 48 to < 72 h), sex (male; female or indeterminate), trial site, multiple births, mode of respiratory support at randomisation ((1) invasive ventilation (by an endotracheal tube); or (2) non-invasive respiratory support through, nasal CPAP, nasal ventilation, humidified high flow nasal cannula therapy or, low flow oxygen ≥ 1.1 L/min; or (3) receiving no mechanical ventilation, or pressure support (in room air, or low flow oxygen < 1.1 L/min, or ambient oxygen)), and receiving inotropes or not at the time of randomisation. Babies of multiple births will be randomised individually.

The Senior Trials Programmer at the NPEU CTU will write the randomisation programme and hold the treatment allocation codes. If necessary, the code may be broken for a single baby at the request of the site principal investigator (PI) or clinician in charge of the baby.

### Data collection schedule

Data will be collected using paper case report forms (CRFs) at the centres and entered into the study’s OpenClinica electronic database by NPEU CTU staff.

The CRFs to be completed are as follows:

- Form 1: Trial entry
- Form 2: Trial medication
- Form 3: ECHO results
- Form 4: 36-week form
- Form 5: Open-label treatment of PDA
- Form 6: Baby outcomes
- Form 6a: Necrotising enterocolitis report form
- Form 7: Baby withdrawal form
- Form 8a: SAE report form
- Form 8b: SAE assessment form
- Form 8c: SAE processing form
- Form 9a: Incident and deviation form
- Form 9b: Incident deviation and serious breach form

Additionally, a 2-year follow-up questionnaire will be sent to parents of participating infants when they reach 2 years of age. These will be completed by the parents, either on the paper questionnaire or an online form, followed by data entry into the study’s OpenClinica database by NPEU CTU staff, where necessary.

Table 3 summarises the schedule of data collection and trial assessments.

**Table 3:** Trial assessments

Procedure	Baby hospitalisation					
	Screening^**a**^	Trial entry and treatment (days 1−3)	Up to 7 days after trial medication	3 weeks of age	36 weeks of PMA	Discharge
**Demography**^**g**^		**✓**				**✓**
**Echocardiogram/colour Doppler**^**f**^	**✓**			**✓**		
**Confirmation of eligibility**	**✓**					
**Consent**		**✓**				
**Randomisation**^**b**^		**✓**				
**Ibuprofen/placebo dosing**^**c**^		**✓**				
**IVH/PVL ultrasound scans**^**h**^			**✓**		**✓**	
**NEC**						**✓**
**Oxygen reduction test**					**✓**	
**SAEs**^**d**^		**✓**	**✓**			
**Concomitant medication**^**e**^	**✓**	**✓**		**✓**	**✓**	**✓**

^a^Screening assessments to be completed sufficiently in advance to enable randomisation and dosing within 72 h of birth. If consent cannot be obtained before echocardiographic evaluation for eligibility, echocardiographic assessment should continue, and consent obtained when possible if a baby is deemed eligible

^b^Randomisation to be completed sufficiently in advance to enable dosing within 72 h of birth

^c^Initial trial drug administrations to be given soon after randomisation, after 6 h of age and within 72 h of birth. Subsequent doses to be administered 24 h after the initial dose

^d^Only adverse events which are serious will be recorded from first dose until 7 days after trial medication. Only unforeseeable SAEs will be reported

^e^Concomitant medications to be recorded only in relation to unforeseeable SAEs. In the event of an unforeseeable SAE all concomitant medication, including medication given to the baby’s mother, 7 days prior to the onset of the event to the time of its resolution must be recorded on the SAE form

^f^An echocardiogram scan will be performed when the baby reaches around 3 weeks of age (range of 18–24 days) or at hospital discharge if discharged earlier

^g^Demography and medications will be assessed through the PARCA-R and other questionnaires

^h^If a baby transfers from the recruiting site to a continuing care site for on-going care details of any scan would be helpful

### Interim analyses and stopping rules

A Data Monitoring Committee (DMC), independent of the applicants and of the Trial Steering Committee (TSC), will review the progress of the trial at least annually and provide advice on the conduct of the trial to the TSC and (via the TSC) to the HTA. The committee will periodically review trial progress and outcomes as well as secondary outcomes (e.g. death, severe IVH).

Interim analyses will be supplied, in strict confidence, to the DMC, as frequently as the Chair requests. The DMC will aim to meet in person at least annually, or more often as appropriate. At the request of the DMC, interim meetings, in person or by teleconference, will be organised. Major trial issues may need to be dealt with between meetings, by phone or by email.

The DMC will be blinded to treatment allocations. The trial statistician will bring a sealed envelope containing the treatment allocations (provided by Head of Trials Programming) that can be opened to break the blind, if considered necessary.

In the light of interim data and other evidence from relevant studies, the DMC will inform the TSC if, in its view, there is proof beyond reasonable doubt that the data indicate that the trial should be terminated. A decision to inform the TSC of such a finding will in part be based on statistical considerations. Appropriate proof beyond reasonable doubt cannot be specified precisely. A difference of at least 3 standard errors in the interim analysis of a major endpoint may be needed to justify halting or modifying the study prematurely.

### Trial reporting

The trial will be reported according to the principles of the CONSORT statement [8]. Analysis of the short-term health outcomes will be conducted after follow-up to discharge has been completed for the last baby to be discharged.

## Protocol non-compliances

A protocol non-compliance is defined as a failure to adhere to the protocol such as the wrong intervention being administered, incorrect data being collected and documented, errors in applying inclusion/exclusion criteria or missed follow-up visits due to error.

All protocol non-compliances will be listed in the final report. Non-compliances are defined below.

### Major

The following are pre-defined major protocol non-compliances with a direct bearing on the primary outcome:

- Data considered to be fraudulent

### Minor

The following will be defined as minor protocol non-compliances:

#### Participants randomised in error

These include infants:

- Who are < 23 weeks or ≥ 29 weeks of gestation
- Who are ≥ 72 h old
- With a PDA < 1.5 mm in diameter OR who does not have unrestrictive pulsatile left to right flow or, growing pattern with right to left flow of 30% or more
- Who have clinical or echocardiography evidence of pulmonary hypertension
- Where written informed consent has not been obtained from the parent(s)
- With a severe congenital anomaly
- With contraindications to the use of ibuprofen
- Who have had indomethacin, ibuprofen, or paracetamol administered after birth

### Treatment non-compliances

These include infants who:

- Do not receive the allocated intervention. These include infants who were allocated ibuprofen, and instead received placebo, and vice versa.
- Do not receive the correct number of doses. These include infants who received less than 3 doses of the trial medication.
- Do not receive medication at the correct time. These include infants who received their first dose later than 72 h after birth, or received their 2^nd^ or 3^rd^ dose outside the specified dosing window (< 18 h or > 72 h between doses 1 and 2, or doses 2 and 3; or dose 3 completed > 7 days after first dose administered).
- Received open-label treatment without meeting the criteria. These include infants who received open-label treatment but did not meet the defined criteria for doing so:

1. Clinical findings of inability to wean on ventilator (ventilated for at least 7 days continuously) AND inability to wean oxygen

OR

Persistent hypotension and/or pulmonary haemorrhage and/or signs of cardiac failure

2. Echocardiographic findings of a large PDA (PDA ≥ 2.0 mm with pulsatile flow)

AND

Hyperdynamic circulation and/or ductal steal (please refer to Baby-OSCAR ECHO workbook).

#### Trial procedure non-compliances

- ECHO not done around 3 weeks (18–24 days) of age
- Oxygen reduction test not done when baby was eligible

Protocol non-compliances will be reported in a process outcomes table.

## Analysis populations

### Post-randomisation exclusions

Exclusions to the analysis post-randomisation are defined as any of the following:

- Infants for whom a written consent form from the parent(s) was not received
- Infants for whom consent to use their data was withdrawn by the parent(s)
- Infants for whom an entire record of fraudulent data was detected (should fraudulent data be detected, consideration will be given to excluding all data for the site where such data were found).

### Population definitions

#### Intention to treat population

The intention to treat (ITT) population will be all infants randomised, excluding post-randomisation exclusions.

#### Interim analysis population

Different denominators will be used in the interim analysis:

- Baseline data will be reported for all trial participants with available data, excluding known post-randomisation exclusions.
- Outcome data will be reported for babies who can be described as ‘completers’, i.e. all trial participants with available data who have died or been discharged home, excluding post-randomisation exclusions.
- Process outcomes will be reported for all trial participants with available data, excluding known post-randomisation exclusions.
- Safety data will be reported for all trial participants who received at least one dose of the study drug.

#### Safety population

All infants randomised who received at least one dose of the study drug.

## Descriptive analyses

### Representativeness of trial population and participant throughput

The flow of participants through each stage of the trial will be summarised by randomised group using a figure presenting the flow of participants. This will describe the numbers of infants:

- Assessed for eligibility
- Eligible
- Randomised
- Allocated to ibuprofen
  - Did not receive allocated treatment (with reasons)
  - Randomised in error
- Allocated to placebo
  - Did not receive allocated treatment (with reasons)
  - Randomised in error
- Withdrawals
- Included in safety population
- Post-randomisation exclusions (with reasons)
- Included in the ITT population

### Baseline comparability of randomised groups

Baseline demographic and clinical characteristics at trial entry will be described for all infants and their mothers in the ITT population by randomised group. The following characteristics will be described:

#### Mother’s baseline characteristics

- Ethnicity
- Age (years)
- Deprivation index
- Antenatal steroid use (any)
  - < 24 h before birth
  - ≥ 24 h before birth
- Antenatal COX inhibitor use
- Antenatal magnesium sulphate use for neuroprotection

#### Infant’s characteristics at trial entry

- Enrolling centre
- Born in enrolling centre
- Postnatal age at randomisation (hours)
- Gestational age at birth (weeks)
- Mode of delivery
- Forceps or Ventouse used in delivery
- Main cause of preterm birth
- Birth weight (g)
- Birth weight z score
- Head circumference (cm)
- Head circumference z score
- Sex
- Baby is one of a multiple pregnancy
- Sibling enrolled in the study (in multiple pregnancies)
- APGAR score 5 min after birth
- Baby’s worst base excess at first hour after birth
- CRIB II (without temperature) [9]
- Size of PDA
- Mode of respiratory support at randomisation
- Receiving inotropes at randomisation

The number and percentage will be presented for binary and categorical variables. The mean and standard deviation or the median and the interquartile range will be presented for continuous variables, and the range if appropriate. There will be no tests of statistical significance performed nor confidence intervals calculated for differences between randomised groups on any baseline variable.

### Losses to follow-up

Minimal loss to follow-up is expected for the primary outcome since it is a short-term outcome and recorded whilst the baby is in hospital.

## Comparative analyses

Infants will be analysed according to their allocation, regardless of the intervention they received (ITT population). The placebo group will be used as the reference group in all analyses.

Outcomes will be summarised with counts and percentages for categorical variables, means and standard deviations for normally distributed continuous variables, or median and interquartile range for other non-normally distributed continuous or time-to-event variables.

For binary outcomes, risk ratios and confidence intervals will be calculated using log binomial regression, and if a model fails to converge a Poisson regression model with a robust variance estimator will be used. Continuous outcomes will be analysed using linear regression models, with mean differences and confidence intervals presented for approximately normally distributed outcomes. Skewed continuous outcomes will be analysed using quantile regression models, with median differences and confidence intervals presented. Time-to-event outcomes will be analysed using Cox regression and hazard ratios with confidence intervals will be presented.

Analyses will be adjusted for all minimisation factors where technically possible. Centre will be treated as a random effect in the models, and all other factors as fixed effects, including multiple births. Correlation between siblings from multiple births will be accounted for in the adjusted model by nesting ‘multiple’ cluster as a random effect within centre. All factors will be fitted as fixed effects in quantile regression, as random effects cannot be modelled using these methods of analysis. Both crude and adjusted effect estimates will be presented, but the primary inference will be based on the adjusted estimates.

Analysis of secondary outcomes will be clearly delineated from the primary outcomes in any statistical reports produced.

### Detailed definition of outcomes

The detailed derivations of each level of BPD are set out in the Statistical Analysis Plan (Appendix B). Detailed derivations for all other outcomes are described in a separate document.

### Primary analysis

The primary analysis for the primary outcome and all secondary outcomes will be conducted on the modified ITT population, only excluding infants from the analysis of an outcome if their data is missing for that outcome. Minimisation factors will be adjusted for where technically possible, as described above.

### Secondary analyses

A restricted analysis on the primary outcome and its components, excluding infants who received open-label treatment without meeting the specified criteria.

### Pre-specified subgroup analyses

Pre-specified subgroup analyses will use the statistical test of interaction and where appropriate, results will be presented as risk ratios with confidence intervals. We appreciate that the trial is not powered to examine tests of interaction, and therefore interpretations of these will be cautious.

Pre-specified subgroup analyses on the primary outcome and its components will be based on:

- gestational age at birth (23 to 23^+6^ weeks; 24 to 24^+6^ weeks; 25 to 25^+6^ weeks; 26 to 26^+6^ weeks; 27 to 27^+6^ weeks; 28 to 28^+6^ weeks)
- size of the PDA (1.5 mm to < 2.0 mm; 2.0 mm to < 3.0 mm; ≥ 3.0 mm)
- mode of respiratory support at randomisation (invasive ventilation (by an endotracheal tube); non-invasive respiratory support through nasal CPAP, nasal ventilation, humidified high flow nasal cannula therapy or, low flow oxygen ≥ 1.1 L/min; or receiving no mechanical ventilation, or pressure support (in room air, or low flow oxygen < 1.1 L/min, or ambient oxygen)).

A further pre-specified subgroup analysis on NEC Bell stage II and above will be conducted by size of the PDA.

### Sensitivity analyses

No sensitivity analyses have been specified.

### Significance levels and adjustment of p-values for multiplicity

95% confidence intervals will be used for all pre-specified outcome comparisons, including subgroup analyses. Due to the large number of secondary outcomes, a subset of outcomes has been pre-specified on which statistical inferences will be made (see Secondary short-term outcomes above).

### Missing data

Missing data will be described, for example, by presenting the number of individuals in the missing category. Missing data as a result of babies being lost to follow-up is expected to be minimal for short-term outcomes.

### Statistical software employed

The statistical software Stata/SE will be used for all analyses.

## Safety data analysis

Serious adverse events (SAEs) will be listed by allocation.

In addition, the following foreseeable SAEs occurring within seven days after trial medication is completed will be reported by trial arm:

- Anaemia requiring transfusion
- Clinically significant intracranial abnormality on cranial ultrasound scan—intracranial haemorrhage or white matter injury
- Coagulopathy requiring treatment
- Culture proven sepsis
- Death (unless unforeseeable in this population)
- Fluid retention
- Gastrointestinal bleeding
- Haematuria
- Haemothorax
- High blood creatinine level (defined as > 100 μmol/L)
- Hyperbilirubinaemia necessitating exchange transfusion
- Hyperglycaemia
- Hypoglycaemia
- Hypotension treated with inotropes
- Impaired renal function (urine output < 0.5 ml/kg/h, and or serum creatinine > 100 μmol/L)
- Low serum sodium level/hyponatraemia (defined as sodium < 130 mmol/L)
- Necrotising enterocolitis
- Neutropenia (defined as < 1.0 mmol/L)
- Pneumothorax requiring treatment
- Pulmonary hypertension requiring treatment with pulmonary vasodilator
- Respiratory failure
- Seizures requiring treatment
- Significant pulmonary haemorrhage
- Spontaneous intestinal perforation
- Thrombocytopenia

## Additional exploratory analysis

Any analyses not specified in the analysis protocol will be exploratory in nature and will be documented in a separate statistical analysis plan. Any post hoc analysis requested by the oversight committees, a journal editor or referees will be labelled explicitly as such.

## Deviation from analysis described in protocol

None yet.

## Supplementary Information

**Additional file 1: Appendix A.** – Baby-OSCAR Dummy Tables v1.0.pdf

**Additional file 2: Appendix B.** – Baby-OSCAR SAP v1.0.pdf

## Data Availability

Information and files related to data management, the Trial Master File and Statistical Master File are held electronically on a secure server at the NPEU CTU.

## Abbreviations

AE: Adverse event
BPD: Bronchopulmonary dysplasia
CI: Confidence interval
CONSORT: Consolidated standards of reporting trails
COX: Cyclo-oxygenase
CPAP: Continuous Positive Airway Pressure
CRIB II: Clinical risk index for babies score II
CRF: Case report form
CTU: Clinical trials unit
DMC: Data monitoring committee
ECHO: Echocardiography
FiO2: Fraction of inspired oxygen
HTA: Health technology assessment
IMP: Investigational Medicinal Product
ITT: Intention to treat
IVH: Intraventricular haemorrhage
NEC: Necrotising enterocolitis
NHS: National Health Service
NIHR: National Institute for Health Research
NNU: Neonatal unit
NPEU: National Perinatal Epidemiology Unit
PDA: Patent ductus arteriosus
PI: Principal investigator
PMA: Postmenstrual age
PVL: Periventricular leukomalacia
RCT: Randomised controlled trial
ROP: Retinopathy of prematurity
SAE: Serious adverse event
SAP: Statistical analysis plan
SmPC: Summary of Product Characteristics
TSC: Trail steering committee
UK: United Kingdom

## References

[1] Gupta S (2021). Study protocol: baby-OSCAR trial: Outcome after Selective early treatment for Closure of patent ductus ARteriosus in preterm babies, a multicentre, masked, randomised placebo-controlled parallel group trial. BMC Pediatr.

[2] Gamble C, Krishan A, Stocken D, Lewis S, Juszczak E, Doré C, Williamson PR, Altman DG, Montgomery A, Lim P, Berlin J, Senn S, Day S, Barbachano Y, Loder E (2017). Guidelines for the content of statistical analysis plans in clinical trials. JAMA.

[3] Noori S, McCoy M, Friedlich P, Bright B, Gottipati V, Seri I, Sekar K (2009). Failure of ductus arteriosus closure is associated with increased mortality in preterm infants. Pediatrics.

[4] Su BH, Lin HC, Chiu HY, Hsieh HY, Chen HH, Tsai YC (2008). Comparison of ibuprofen and indometacin for early-targeted treatment of patent ductus arteriosus in extremely premature infants: a randomised controlled trial. Arch Dis Child Fetal Neonatal Ed.

[5] Oncel MY, Yurttutan S, Degirmencioglu H, Uras N, Altug N, Erdeve O, Dilmen U (2013). Intravenous paracetamol treatment in the management of patent ductus arteriosus in extremely low birth weight infants. Neonatology.

[6] Schmidt B, Davis P, Moddemann D, Ohlsson A, Roberts RS, Saigal S, Solimano A, Vincer M, Wright LL (2001). Long-term effects of indomethacin prophylaxis in extremely-low-birth-weight infants. N Engl J Med.

[7] Neonatal Survey Database from the Trent Region. 2010 []; Available from: http://www.le.ac.uk/departments/health-sciences/research/timms/projects/tns.

[8] Schulz KF, Altman DG, Moher D, for the CONSORT Group (2010). CONSORT 2010 statement: updated guidelines for reporting parallel group randomised trials. BMJ.

[9] Parry G, et al. CRIB II: an update of the clinical risk index for babies score. Lancet. 2003;361(9371):1789–91. 10.1016/S0140-6736(03)13397-1 12781540

