# Correction to: Baby-OSCAR: Outcome after Selective early treatment for Closure of patent ductus ARteriosus in preterm babies—a statistical analysis plan for short-term outcomes

**DOI:** 10.1186/s13063-021-05409-z

**Published:** 2021-09-09

**Authors:** Jennifer L. Bell, Samir Gupta, Edmund Juszczak, Pollyanna Hardy, Louise Linsell

**Affiliations:** 1 University of Oxford, Oxford, UK; 2 Durham University, Durham, UK; 3 University of Nottingham, Nottingham, UK; 4 University of Birmingham, Birmingham, UK

**Correction to: Trials 22, 368 (2021)**

**https://doi.org/10.1186/s13063-021-05324-3**

Following the publication of the original article [1], It has come to the authors’ attention that some wording was changed prior to publication of the Statistical Analysis Plan from the original SAP which will be used in their analysis of data.

The original wording used in the SAP was:

1. Inability to wean on ventilator (ventilated for at least 7 days continuously) and inability to wean oxygen, or
2. Persistent hypotension/pulmonary haemorrhage/signs of cardiac failure
  AND
3. Echocardiographic findings of a large PDA (PDA ≥ 2.0 mm with pulsatile flow) AND hyperdynamic circulation or ductal steal (refer to Baby-OSCAR ECHO workbook).

However, the wording in this section in the published SAP was:

1. Clinical findings on inability to wean on ventilator (ventilated for at least 7 days continuously) AND inability to wean oxygen; OR Persistent hypotension and/or pulmonary haemorrhage and/or signs of cardiac failure
  AND
2. Echocardiographic findings of a large PDA (PDA ≥ 2.0 mm with pulsatile flow)
  AND
  Hyperdynamic circulation and/or ductal steal (refer to Baby-OSCAR ECHO workbook)

The original article has been corrected.
