# Supplementary material for: Baby-OSCAR: Outcome after Selective early treatment for Closure of patent ductus ARteriosus in preterm babies—a statistical analysis plan for short-term outcomes
Source: Trials. 2021 May 26;22:368. doi: 10.1186/s13063-021-05324-3 (PMC8157743; doi:10.1186/s13063-021-05324-3)
Supplement: Supplementary file 1 — Additional file 1: Appendix A. – Baby-OSCAR Dummy Tables v1.0.pdf [file 13063_2021_5324_MOESM1_ESM.pdf]

Outcome after Selective Early Treatment for Closure of Patent Ductus ARteriosus in Preterm Babies

Dummy Tables
Short term outcomes

[STRICTLY CONFIDENTIAL]

Version x.x, dd mmm yyyy

Template version 1.0, 19 February 2021

Author: Jennifer Bell (Trial statistician, NPEU CTU)

Reviewers: Dr Louise Linsell (Lead statistician 2017 to present, NPEU CTU)
Prof Samir Gupta (Chief Investigator)

Contents

List of abbreviations 3

Figure 1: Consort Flow Chart 4

Table 1: Mother's baseline characteristics 5

Table 2: Infant's characteristics at trial entry 6

Table 3: Primary outcome 9

Table 4a: Secondary short-term outcomes (tested) 10

Table 4a: Secondary short-term outcomes (untested) 12

Table 5: Safety 14

Table 6: Unforeseeable serious adverse events by allocation 15

Table 7: Process outcomes 16

Table 8a: Subgroup analysis: Primary outcome and components 18

Table 8b: Subgroup analysis: NEC Bell Stage II and above 20

Figure 2: Subgroup analysis forest plot 20

Table 9: Secondary analysis: Restricted analysis excluding infants who received open label treatment without meeting the specified criteria 21

DOCUMENT HISTORY 22

List of abbreviations

AE	Adverse event
BPD	Bronchopulmonary dysplasia
CI	Confidence interval
CONSORT	Consolidated standards of reporting trails
COX	Cyclo-oxygenase
cm	Centimetre
CPAP	Continuous Positive Airway Pressure
CRIB II	Clinical risk index for babies score II
CRF	Case report form
CTU	Clinical trials unit
DMC	Data monitoring committee
ECHO	Echocardiography
FiO2	Fraction of inspired oxygen
g	Gram
IMP	Investigational Medicinal Product
IVH	Intraventricular haemorrhage
kg	Kilogram
L	Litre
M	Metre
mg	Milligram
min	Minute
ml	Millilitre
mm	Millimetre
mmol	Millimole
µmol	Micromole
NEC	Necrotising enterocolitis
NNU	Neonatal unit
NPEU	National Perinatal Epidemiology Unit
PDA	Patent ductus arteriosus
PMA	Postmenstrual Age
PVL	Periventricular leukomalacia
ROP	Retinopathy of prematurity
SAE	Serious adverse event

Figure 1: Consort Flow Chart

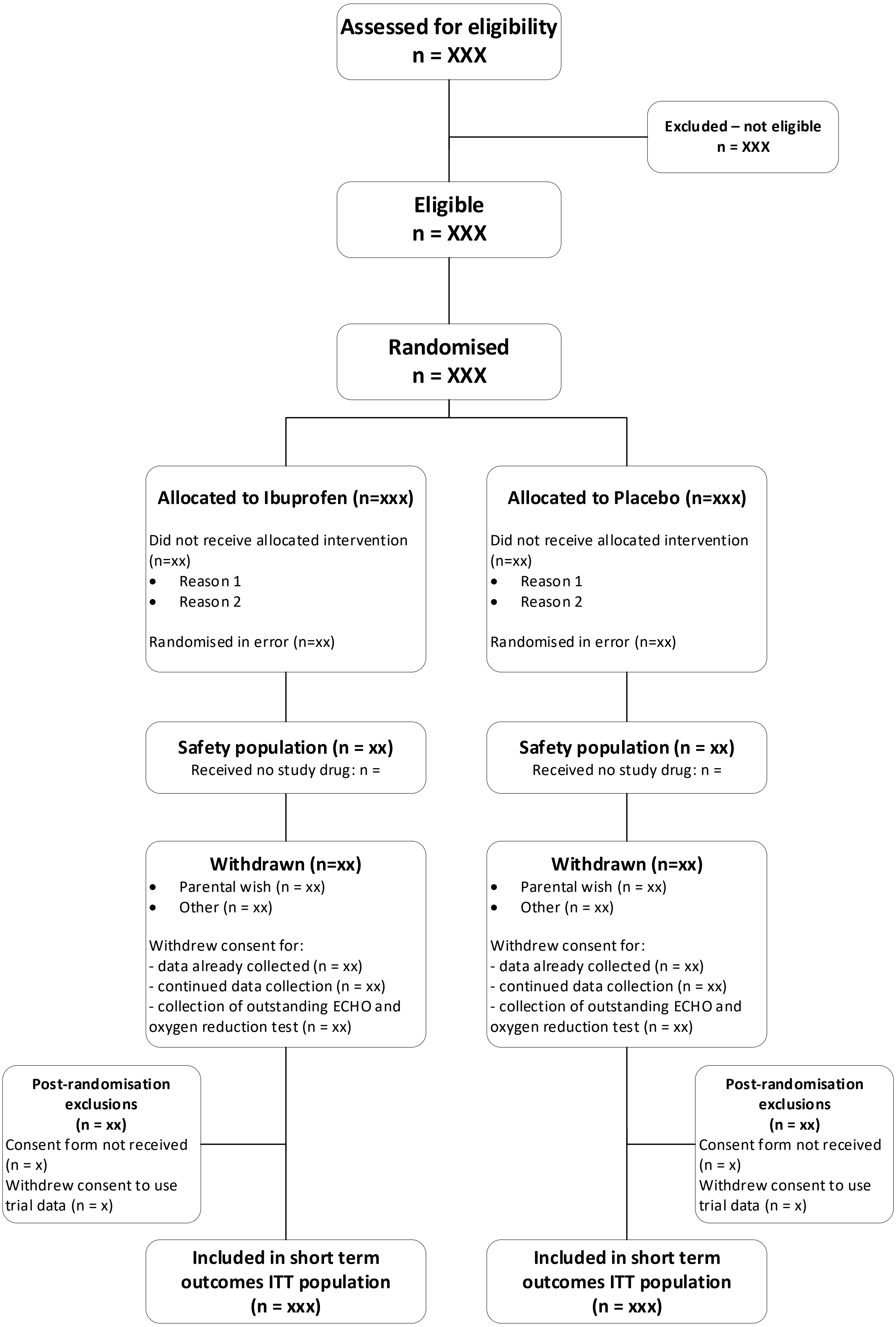

Table 1: Mother's baseline characteristics

	Ibuprofen (n = xxx)	Placebo (n = xxx)
**Mother's ethnicity, n (%)**		
White		
Asian		
Black		
Mixed		
Other		
Missing		
**Mother's age (years), mean (SD)**		
Median [IQR]		
(Min to max)		
Missing		
**Deprivation Index, n (%)**		
1 (Least deprived)		
2		
3		
4		
5 (Most deprived)		
Wales – not defined		
Missing		
**Antenatal steroid use, n (%)**		
Any		
< 24 hours before birth		
≥ 24 hours before birth		
Missing		
**Antenatal COX inhibitor use, n (%)**		
Missing		
**Antenatal magnesium sulphate use for neuroprotection, n (%)**		
Missing		

Table 2: Infant's characteristics at trial entry

* denotes minimisation factor

	Ibuprofen (n = xxx)	Placebo (n = xxx)
**Enrolling centre*, n (%)**		
1		
2		
3		
4		
5		
6		
7		
8		
9		
10		
11		
12		
13		
14		
15		
16		
17		
18		
19		
20		
21		
22		
23		
24		
25		
26		
27		
28		
29		
30		
**Born in enrolling centre, n (%)**		
**Postnatal age at randomisation (hours)*, mean (SD)**		
Median [IQR]		
(Min to max)		
< 12 hours, n (%)		
12 to < 24 hours, n (%)		
24 to < 48 hours, n (%)		
48 to < 72 hours, n (%)		
**Gestational age at birth (weeks)*, n (%)**		
Median [IQR]		
(Min to max)		
23 to < 24 weeks		
24 to < 25 weeks		

	Ibuprofen (n = xxx)	Placebo (n = xxx)
25 to < 26 weeks		
26 to < 27 weeks		
27 to < 28 weeks		
28 to < 29 weeks		
**Mode of delivery, n (%)**		
Vaginal birth – cephalic		
Vaginal birth – breech		
Caesarean section before onset of labour		
Caesarean section after onset of labour		
Missing		
**Forceps or Ventouse used in delivery, n (%)**		
Missing		
**Main cause of preterm birth, n (%)**		
Preterm prelabour rupture of membranes (PPROM)		
Preterm labour (without PROM)		
APH		
PIH (+/- APH)		
Other maternal illness[1]		
Obstetric intervention for fetal reasons		
Obstetric intervention for maternal reasons		
Other		
Missing		
**Birth weight (g), N**		
Mean (SD)		
Median [IQR]		
(Min to max)		
Missing		
**Birth weight z score, N**		
Mean (SD)		
Missing		
**Head circumference (cm), mean (SD)**		
Median [IQR]		
(Min to max)		
Missing		
**Head circumference z score, mean (SD)**		
Missing		
**Sex*, n (%)**		
Male		
Female		
**Baby is one of a multiple pregnancy*, n (%)**		
Missing		
**Sibling enrolled in the study (in multiple pregnancies), n (%)**		
Missing		

[1] Any pregnancy where the main reason for preterm delivery was a maternal problem such as infection, renal disease or pre-pregnancy diabetes, hypertension or trauma.

	Ibuprofen (n = xxx)	Placebo (n = xxx)
**APGAR score 5 minutes after birth, N**		
Mean (SD)		
Median [IQR]		
(Min to max)		
< 7 at 5 minutes, n (%)		
≥ 7 at 5 minutes, n (%)		
Missing		
**Baby's worst base excess at first hour after birth[2], N**		
Mean (SD)		
Median [IQR]		
(Min to max)		
Missing		
**CRIB II (without temperature), N**		
Mean (SD)		
Median [IQR]		
(Min to max)		
Missing		
**Size of PDA*, N**		
Mean (SD)		
Median [IQR]		
(Min to max)		
≥ 1.5 mm and < 2.0 mm, n (%)		
≥ 2.0 mm and < 3.0 mm, n (%)		
≥ 3.0 mm, n (%)		
**Mode of respiratory support at randomisation*, n (%)**		
Invasive ventilation (by ET tube)		
Non-invasive respiratory support only[3]		
Receiving no mechanical ventilation or pressure support[4]		
Missing		
**Receiving inotropes*, n (%)**		
Missing		

[2] 'highest' negative
[3] Nasal CPAP, nasal ventilation, humidified high flow nasal cannula therapy, or low flow oxygen ≥ 1.1L/min
[4] In room air, low flow oxygen < 1.1L/min, or ambient oxygen)

Table 3: Primary outcome

	Ibuprofen (n = xxx)	Placebo (n = xxx)	Unadjusted risk ratio (95% CI)	Adjusted risk ratio (95% CI)	p-value
**Death by or moderate/severe BPD at 36 weeks' postmenstrual age, n (%)**<br>Missing			RR x.xx (x.xx to x.xx)	RR x.xx (x.xx to x.xx)	p

Table 4a: Secondary short-term outcomes (tested)

	Ibuprofen (n = xxx)	Placebo (n = xxx)	Unadjusted effect estimate (95% CI)	Adjusted effect estimate (95% CI)	p-value
**Death by 36 weeks of postmenstrual age, n (%)**			RR x.xx (x.xx to x.xx)	RR x.xx (x.xx to x.xx)	p
Missing					
**Moderate or severe BPD at 36 weeks of postmenstrual age, n (%)**			RR x.xx (x.xx to x.xx)	RR x.xx (x.xx to x.xx)	p
Missing					
**Any intraventricular haemorrhage (IVH), n (%)**					
Grade I/II without ventricular dilatation					
Severe IVH (grade III/IV)[5]			RR x.xx (x.xx to x.xx)	RR x.xx (x.xx to x.xx)	p
Missing					
**Cystic PVL, n (%)**			RR x.xx (x.xx to x.xx)	RR x.xx (x.xx to x.xx)	p
Missing					
**Baby treated for Retinopathy of prematurity (ROP)[6], n (%)**			RR x.xx (x.xx to x.xx)	RR x.xx (x.xx to x.xx)	p
Missing					
**Worst stage of ROP in either eye, n (%)**			-	-	
Stage I					
Stage II					
Stage II + disease					
Stage III					
Stage III + disease					
Stage IV					
Stage V					
**AP-ROP in either eye, n (%)**			-	-	
Missing					
**Significant pulmonary haemorrhage[7], n (%)**			RR x.xx (x.xx to x.xx)	RR x.xx (x.xx to x.xx)	p
Missing					
**Diagnosed with pulmonary hypertension, n (%)**			-	-	
Clinically					
ECHO					
Missing					
**Treated for pulmonary hypertension with pulmonary vasodilator, n (%)**			RR x.xx (x.xx to x.xx)	RR x.xx (x.xx to x.xx)	p
Nitric oxide					
Other					
Missing					
**NEC Bell stage II and above[8], n (%)**			RR x.xx (x.xx to x.xx)	RR x.xx (x.xx to x.xx)	p
Missing					

[5] With ventricular dilatation or intraparenchymal abnormality
[6] In at least one eye
[7] Fresh blood in endotracheal tube with increase in respiratory support
[8] Confirmed by radiography and/or histopathology

	Ibuprofen (n = xxx)	Placebo (n = xxx)	Unadjusted effect estimate (95% CI)	Adjusted effect estimate (95% CI)	p-value
**Closed or non-significant PDA (< 1.5mm) at around 3 weeks[9] of age, confirmed by ECHO, n (%)**			RR x.xx (x.xx to x.xx)	RR x.xx (x.xx to x.xx)	p
Missing					
**PDA ≥ 1.5mm at around 3 weeks'[9], not treated medically or by surgical closure, n (%)**			RR x.xx (x.xx to x.xx)	RR x.xx (x.xx to x.xx)	p
Missing					
**Open-label treatment of a symptomatic PDA by surgical treatment, n (%)**			RR x.xx (x.xx to x.xx)	RR x.xx (x.xx to x.xx)	p
Missing					
**Discharge home on oxygen, n (%)**			RR x.xx (x.xx to x.xx)	RR x.xx (x.xx to x.xx)	p
Missing					
**Weight gain: a change in z score between birth and discharge, mean (SD)**			MD x.xx (x.xx to x.xx)	MD x.xx (x.xx to x.xx)	p
(Min to max)					
Missing					

[9] Between 18 and 24 days of age

Table 4a: Secondary short-term outcomes (untested)

	Ibuprofen (n = xxx)	Placebo (n = xxx)
**Severity of BPD at 36 weeks of postmenstrual age, n (%)**		
No BPD		
Mild BPD		
Moderate BPD		
Severe BPD		
Missing		
**Hydrocephalus (ventricular index > 4 mm above 97th centile), n (%)**		
Missing		
**Non-cystic PVL, n (%)**		
Missing		
**Other white matter injury[10], n (%)**		
Missing		
**NEC requiring surgery, n (%)**		
Missing		
**Gastrointestinal bleeding (leading to investigation or clinical treatment) within 7 days of first dose, n (%)**		
Missing		
**Spontaneous intestinal perforation, n (%)**		
Missing		
Surgical management undertaken		
Missing		
**Medical open-label treatment of a symptomatic PDA with a COX inhibitor, n (%)**		
**Administration of inotropic support, n (%)**		
Missing		
**Duration of inotropic support (days), median [IQR]**		
(Min to max)		
Missing		
**Diuretics used for management of PDA[10], n (%)**		
Missing		
**Total duration of respiratory support (days), median [IQR]**		
(Min to max)		
Missing		
**Invasive ventilation through an endotracheal tube, n (%)**		
Duration (days), median [IQR]		
(Min to max)		
Missing		
**Non-invasive respiratory support through nasal CPAP, nasal ventilation, humidified high flow nasal cannula therapy, or low flow oxygen ≥ 1.1 L/min, n (%)**		
Duration (days), median [IQR]		
(Min to max)		

[10] For descriptive purposes only

Missing<br>**Ambient or low-flow oxygen (< 1.1 L/min)[11], n (%)**<br>Duration (days), median [IQR]<br>(Min to max)<br>Missing<br>**Duration of initial hospitalisation (birth to discharge home) , median [IQR]**<br>(Min to max)<br>Missing<br>**Postnatal steroid use for chronic lung disease, n (%)**<br>Missing<br>**Head circumference: a change in z score between birth and discharge, mean (SD)**<br>Missing		

[11] For descriptive purposes only

Table 5: Safety

	Ibuprofen (n = xxx)	Placebo (n = xxx)
**Tolerance of ibuprofen treatment within foreseeable SAE reporting range, n (%)**		
Anaemia requiring transfusion		
Clinically significant intracranial abnormality on cranial ultrasound scan – intracranial haemorrhage or white matter injury		
Coagulopathy requiring treatment		
Culture proven sepsis		
Death		
Fluid retention		
Gastrointestinal bleeding		
Haematuria		
Haemothorax		
High blood creatinine level[12]		
Hyperbilirubinemia necessitating exchange transfusion		
Hypotension treated with inotropes		
Impaired renal function[13]		
Low serum sodium level/hyponatremia[14]		
Necrotising enterocolitis (stage IIA and above)		
Neutropenia[15]		
Pneumothorax requiring treatment		
Seizures requiring treatment		
Significant pulmonary haemorrhage		
Spontaneous intestinal perforation		
Thrombocytopenia		

[12] > 100 µmol/L
[13] Urine output < 0.5 mL/kg/hour, and or serum creatinine > 100µmol/L
[14] Sodium < 130 mmol/L
[15] < 1.0 mmol/L

Table 6: Unforeseeable serious adverse events by allocation

SAE number	Treatment allocation	Centre ID	Description	Severity	Causality	Action taken	Outcome	Related
1	x	x	xxxxx	xxxxx	xxxxx	xxxxx	xxxxx	Yes/No
2	x	x	xxxxx	xxxxx	xxxxx	xxxxx	xxxxx	Yes/No
3	x	x	xxxxx	xxxxx	xxxxx	xxxxx	xxxxx	Yes/No
4	x	x	xxxxx	xxxxx	xxxxx	xxxxx	xxxxx	Yes/No

Table 7: Process outcomes

	Ibuprofen (n = xxx)	Placebo (n = xxx)
**Did not receive allocated intervention, n (%)**		
**Incomplete trial medications, n (%)**		
Missing		
**Doses received, n (%)**		
0		
1		
2		
3		
Missing		
**Stopped early[16] reason**		
Parental decision, n/N (%)		
Clinician decision, n/N (%)		
Died, n/N (%)		
Other, n/N (%)		
Missing		
**Time to first dose (hours), median [IQR]**		
(Min to max)		
Missing		
**Postmenstrual age at first dose (hours), median [IQR]**		
(Min to max)		
0 to <24 hours, n (%)		
24 to <48 hours, n (%)		
48 to <72 hours, n (%)		
≥ 72 hours, n (%)		
Missing		
**Received 2nd or 3rd dose outside of dosing window, n/N (%)**		
<18 hrs between doses 1 and 2		
>72 hrs between doses 1 and 2		
<18 hrs between doses 2 and 3		
>72 hrs between doses 2 and 3		
Dose 3 >7 days after dose 1		
**ECHO not done around 3 weeks of age, n (%)**		
Lack of personnel		
Reason 2		
Reason 3		
**Non-symptomatic open-label treatment[17], n (%)**		
Medical treatment		
Surgical ligation of PDA		
Other		
**Oxygen reduction test not done when baby was eligible, n (%)**		
Missing		

[16] Less than 3 doses
[17] Those who did not meet the criteria for open-label treatment and still received it

	Ibuprofen (n = xxx)	Placebo (n = xxx)
**Study withdrawals, n (%)**		

Table 8a: Subgroup analysis: Primary outcome and components

Data presented are n/N (%)

	Ibuprofen (n = XXX)	Placebo (n = XXX)	Adjusted effect estimate (95% CI)	Interaction p-value
**Gestational age at birth**				
**Primary outcome**				p
23 to 23^{+6} weeks			RR x.xx (x.xx to x.xx)	
24 to 24^{+6} weeks			RR x.xx (x.xx to x.xx)	
25 to 25^{+6} weeks			RR x.xx (x.xx to x.xx)	
26 to 26^{+6} weeks			RR x.xx (x.xx to x.xx)	
27 to 27^{+6} weeks			RR x.xx (x.xx to x.xx)	
28 to 28^{+6} weeks			RR x.xx (x.xx to x.xx)	
**Death by 36 weeks' PMA**				p
23 to 23^{+6} weeks			RR x.xx (x.xx to x.xx)	
24 to 24^{+6} weeks			RR x.xx (x.xx to x.xx)	
25 to 25^{+6} weeks			RR x.xx (x.xx to x.xx)	
26 to 26^{+6} weeks			RR x.xx (x.xx to x.xx)	
27 to 27^{+6} weeks			RR x.xx (x.xx to x.xx)	
28 to 28^{+6} weeks			RR x.xx (x.xx to x.xx)	
**Moderate or severe BPD at 36 weeks' PMA**				p
23 to 23^{+6} weeks			RR x.xx (x.xx to x.xx)	
24 to 24^{+6} weeks			RR x.xx (x.xx to x.xx)	
25 to 25^{+6} weeks			RR x.xx (x.xx to x.xx)	
26 to 26^{+6} weeks			RR x.xx (x.xx to x.xx)	
27 to 27^{+6} weeks			RR x.xx (x.xx to x.xx)	
28 to 28^{+6} weeks			RR x.xx (x.xx to x.xx)	
**Size of PDA**				
**Primary outcome**				p
1.5 mm to < 2.0 mm			RR x.xx (x.xx to x.xx)	
2.0 mm to < 3.0 mm			RR x.xx (x.xx to x.xx)	
≥ 3.0 mm			RR x.xx (x.xx to x.xx)	
**Death by 36 weeks' PMA**				p
1.5 mm to < 2.0 mm			RR x.xx (x.xx to x.xx)	
2.0 mm to < 3.0 mm			RR x.xx (x.xx to x.xx)	
≥ 3.0 mm			RR x.xx (x.xx to x.xx)	
**Moderate or severe BPD at 36 weeks' PMA**				p
1.5 mm to < 2.0 mm			RR x.xx (x.xx to x.xx)	
2.0 mm to < 3.0 mm			RR x.xx (x.xx to x.xx)	
≥ 3.0 mm			RR x.xx (x.xx to x.xx)	
**Mode of respiratory support at randomisation**				

	Ibuprofen (n = XXX)	Placebo (n = XXX)	Adjusted effect estimate (95% CI)	Interaction p-value
**Primary outcome**				p
Invasive ventilation (by an endotracheal tube)			RR x.xx (x.xx to x.xx)	
Non-invasive respiratory support through nasal CPAP, nasal ventilation, humidified high flow nasal cannula therapy or, low flow oxygen ≥ 1.1 L/min			RR x.xx (x.xx to x.xx)	
Receiving no mechanical ventilation, or pressure support (in room air, or low flow oxygen < 1.1 L/min, or ambient oxygen)			RR x.xx (x.xx to x.xx)	
**Death by 36 weeks' PMA**				p
Invasive ventilation (by an endotracheal tube)			RR x.xx (x.xx to x.xx)	
Non-invasive respiratory support through nasal CPAP, nasal ventilation, humidified high flow nasal cannula therapy or, low flow oxygen ≥ 1.1 L/min			RR x.xx (x.xx to x.xx)	
Receiving no mechanical ventilation, or pressure support (in room air, or low flow oxygen < 1.1 L/min, or ambient oxygen)			RR x.xx (x.xx to x.xx)	
**Moderate or severe BPD at 36 weeks' PMA**				p
Invasive ventilation (by an endotracheal tube)			RR x.xx (x.xx to x.xx)	
Non-invasive respiratory support through nasal CPAP, nasal ventilation, humidified high flow nasal cannula therapy or, low flow oxygen ≥ 1.1 L/min			RR x.xx (x.xx to x.xx)	
Receiving no mechanical ventilation, or pressure support (in room air, or low flow oxygen < 1.1 L/min, or ambient oxygen)			RR x.xx (x.xx to x.xx)	

Table 8b: Subgroup analysis: NEC Bell Stage II and above

Data presented are n/N (%)

	Ibuprofen (n = XXX)	Placebo (n = XXX)	Adjusted effect estimate (95% CI)	Interaction p-value
**Size of PDA**				p
1.5 mm to < 2.0 mm			RR x.xx (x.xx to x.xx)	
2.0 mm to < 3.0 mm			RR x.xx (x.xx to x.xx)	
≥ 3.0 mm			RR x.xx (x.xx to x.xx)	

Figure 2: Subgroup analysis forest plot

Table 9: Secondary analysis: Restricted analysis excluding infants who received open label treatment without meeting the specified criteria

	Ibuprofen (n = xxx)	Placebo (n = xxx)	Adjusted risk ratio (95% CI)	Adjusted p-value
**Death by or moderate/severe BPD at 36 weeks' postmenstrual age, n (%)**			RR x.xx (x.xx to x.xx)	p
Missing				
**Death by 36 weeks of postmenstrual age, n (%)**			RR x.xx (x.xx to x.xx)	p
Missing				
**Moderate or severe BPD at 36 weeks of postmenstrual age, n (%)**			RR x.xx (x.xx to x.xx)	p
Missing				

DOCUMENT HISTORY

Version number	Date	Author/reviewer	Changes
i	17/07/2014	Clare Nelis	Created
ii	25/02/2015	Jennifer Waiting	Reviewed in line with updated Protocol, added long term and process outcomes
iii	26/02/2015	Jennifer Waiting	Reordered and added endpoints
iv	05/03/2015	Jennifer Waiting	Formatting, queries for reviewers
v	11/03/2015	Jennifer Waiting	Column headings, formatting, amendments from Pollyanna Hardy
vi	11/03/2015	Jennifer Waiting	Changes accepted – ready for review
vii	18/03/2015	Jennifer Waiting	Adherence to protocol outcomes added as requested by CI
viii	19/05/2015	Jennifer Waiting. Reviewed by PMG	Amendments following review at PMG 06/05/15.
ix	10/06/2015	Jennifer Waiting. Reviewed by Samir Gupta.	Amendments to participant flow chart, and minor changes to wording and categories of outcomes.
x	16/06/2015	Jennifer Waiting. Reviewed by Samir Gupta.	Clarifications on long term outcomes received and changes made. Minor changes to wording.
xi	02/07/2015	Jennifer Waiting. Reviewed by CiG 1st July 2015.	Minor rewording and grammatical changes. Added highest qualification level, time to first dose.<br>Amended Main cause of preterm birth options and Mode of delivery.<br>Replaced 'Forceps or ventouse used' with Presentation.
xii	04/11/2015	Jennifer Bell (née Waiting).	Amended 'Main cause of preterm birth' options following SG review of data derivation document.
xiii	24/11/2015	Jennifer Bell. Reviewed by DMC.	Changed 'worse base excess' to 'highest base excess'.<br>Inserted Safety table.<br>Amended table numbers.
xiv	20/09/2016	Jennifer Bell	Amendments in light of protocol and CRF changes
xv	06/10/2016	Jennifer Bell	Further amendments to protocol and CRF, and clarification on derivations
xvi	03/02/2017	Jennifer Bell	Minor amendments with tracked changes
0.17	28/07/2017	Jennifer Bell	Updated to match current CRFs and protocol, in preparation for first analysis
0.18	26/09/2017	Jennifer Bell	Updated following programming for interim analysis
0.19	18/06/2019	Jennifer Bell	Updated for review by CI and DMC
0.20	04/02/2020	Jennifer Bell	Amended CONSORT, subgroup, sensitivity and secondary analyses following review with collaborators. Moved long term outcomes table to separate document. Reformatted.
1.0	19/02/2021	Jennifer Bell	Updated in line with finalised SAP. Removed Sensitivity analyses.
