# Supplementary material for: Baby-OSCAR: Outcome after Selective early treatment for Closure of patent ductus ARteriosus in preterm babies—a statistical analysis plan for short-term outcomes
Source: Trials. 2021 May 26;22:368. doi: 10.1186/s13063-021-05324-3 (PMC8157743; doi:10.1186/s13063-021-05324-3)
Supplement: Supplementary file 2 — Additional file 2: Appendix B. – Baby-OSCAR SAP v1.0.pdf [file 13063_2021_5324_MOESM2_ESM.pdf]

Outcome after Selective Early Treatment for Closure of Patent Ductus ARteriosus in Preterm Babies

Statistical Analysis Plan for Short Term Outcomes

Version 1.0
Date: 8 February 2021

Authors: *Jennifer Bell (Trial statistician, NPEU CTU)*

Reviewers: *Pollyanna Hardy (Senior statistician 2013 - 2017, NPEU CTU)*
Dr Louise Linsell (Lead statistician, 2017 to present, NPEU CTU)
Prof Ed Juszczak (ex-Director, NPEU CTU)
Prof Samir Gupta (Chief investigator)

Protocol version: 7.0

TABLE OF CONTENTS

1 Introduction 5
2 Background information 6
2.1 Rationale 6
2.2 Objectives of the trial 7
2.2.1 Primary objective 7
2.2.2 Secondary objectives 7
2.3 Trial design 8
2.4 Eligibility 8
2.4.1 Inclusion criteria 8
2.4.2 Exclusion criteria 8
2.5 Interventions 8
2.6 Definition of primary and secondary outcomes 9
2.6.1 Primary outcome 9
2.6.2 Secondary short-term outcomes 10
2.6.3 Process outcomes 11
2.7 Hypothesis framework 12
2.8 Sample size & power 12
2.9 Intervention allocation 14
2.10 Data collection schedule 14
2.11 Interim analyses and stopping rules 15
2.12 Trial reporting 16
3 Protocol non-compliances 16
3.1 Major 16
3.2 Minor 16
4 Analysis populations 17
4.1 Post-randomisation exclusions 17
4.2 Population definitions 18
4.2.1 Intention to treat population 18
4.2.2 Interim analysis population 18
4.2.3 Safety population 18
5 Descriptive analyses 18
5.1 Representativeness of trial population and participant throughput 18
5.2 Baseline comparability of randomised groups 19
5.3 Losses to follow-up 20

6 Comparative analyses 20
6.1 Detailed definition of outcomes 20
6.2 Primary analysis 20
6.3 Secondary analyses 21
6.4 Pre-specified subgroup analyses 21
6.5 Sensitivity analyses 21
6.6 Significance levels and adjustment of p-values for multiplicity 21
6.7 Missing data 21
6.8 Statistical software employed 21
7 Safety data analysis 21
8 Additional exploratory analysis 22
9 Deviation from analysis described in protocol 22
10 Appendix A – Detailed derivation of levels of BPD 23
11 References 26
11.1 Trial documents 26
11.2 Other references 26
12 Approval 27
Document history 27

List of Figures

Figure 1: Oxygen reduction test flow chart 10
Figure 2: Sample size per arm vs. Difference in proportions 13

List of Tables

Table 1: Severity-based diagnostic criteria for BPD 9
Table 2: Sample size required by event rates 13
Table 3: Trial assessments 15

List of abbreviations

AE	Adverse event
BPD	Bronchopulmonary dysplasia
CI	Confidence interval
CONSORT	Consolidated standards of reporting trails
COX	Cyclo-oxygenase
cm	Centimetre
CPAP	Continuous Positive Airway Pressure
CRIB II	Clinical risk index for babies score II
CRF	Case report form
CTU	Clinical trials unit
DMC	Data monitoring committee
ECHO	Echocardiography
FiO2	Fraction of inspired oxygen
g	Gram
HTA	Health technology assessment
IMP	Investigational Medicinal Product
ITT	Intention to treat
IVH	Intraventricular haemorrhage
kg	Kilogram
L	Litre
M	Metre
mg	Milligram
min	Minute
ml	Millilitre
mm	Millimetre
mmol	Millimole
µmol	Micromole
NEC	Necrotising enterocolitis
NHS	National Health Service
NIHR	National Institute for Health Research
NNU	Neonatal unit
NPEU	National Perinatal Epidemiology Unit
PDA	Patent ductus arteriosus
PMA	Postmenstrual Age
PVL	Periventricular leukomalacia
RCT	Randomised controlled trial
ROP	Retinopathy of prematurity
SAE	Serious adverse event
SAP	Statistical analysis plan
SmPC	Summary of Product Characteristics
TSC	Trail steering committee
UK	United Kingdom

1 INTRODUCTION

This document details the proposed presentation and analyses for the main analysis reporting the short-term results from the National Institute for Health Research (NIHR) Health Technology Assessment (HTA) programme funded multi-centre, masked, randomised controlled trial, Baby-OSCAR.

The results reported will follow the strategy set out here, which adheres to the guidelines for the content of a statistical analysis plan (SAP). Subsequent analyses of a more exploratory nature will not be bound by this strategy, though they are expected to follow the broad principles laid down here. The principles are not intended to curtail exploratory analysis (e.g. to decide cut-points for categorisation of continuous variables), nor to prohibit accepted practices (e.g. data transformation prior to analysis), but they are intended to establish the rules that will be followed, as closely as possible, when analysing and reporting the trial.

The analysis plan will be available on request when the principal papers are submitted for publication in a journal. Suggestions for subsequent analyses by journal editors or referees, will be considered carefully, and carried out as far as possible in line with the principles of this analysis plan; if reported, the source of the suggestion will be acknowledged.

Any deviations from the statistical analysis plan will be described and justified in the final report of the trial. The analysis should be carried out by an identified, appropriately qualified and experienced statistician, who should ensure the integrity of the data during their processing. Examples of such procedures include quality control and evaluation procedures.

2 BACKGROUND INFORMATION

2.1 Rationale

Baby-OSCAR is a masked, multi-centre randomised placebo-controlled parallel group trial to determine short- and long-term health and economic outcomes of the treatment of a large Patent Ductus Arteriosus (PDA) in extremely preterm babies with ibuprofen within 72 hours of birth.

The main trial follows an internal pilot phase, which has been run to assess the suitability of trial procedures and likelihood of recruitment targets being achieved. The trial aimed to recruit approximately 730 infants in total (including those recruited during the internal pilot phase) from 35 UK tertiary neonatal units (and 5 for the internal pilot phase).

PDA is associated with a number of serious and life-threatening short and long term complications including low blood pressure (hypotension), bleeding in the lungs (pulmonary haemorrhage) and brain (intraventricular haemorrhage (IVH)), systemic complications such as necrotising enterocolitis (NEC), bronchopulmonary dysplasia (BPD), and long term health problems such as neurodevelopmental disability and chronic respiratory problems. The persistence of PDA is associated with an 8-fold rise in neonatal mortality [Noori S et al, 2009]. In addition, as PDA is very common in extreme preterm babies and is associated with a prolonged need for respiratory support and hospitalisation, it places a significant financial burden on the National Health Service (NHS).

Historically, clinicians who have been concerned about the complications associated with a PDA have attempted to close PDAs utilising medical (pharmacological) or surgical treatment. Traditionally, medical treatment is instituted as prophylactic treatment (within 24 hours of birth) or symptomatic treatment (usually 5−7 days after birth). Prophylactic pharmacological treatment of all preterm babies unnecessarily exposes a large proportion of babies to the potentially serious side effects of drug treatment, when their PDA would have closed spontaneously. Symptomatic treatment on the contrary delays treatment while waiting for symptoms to appear and could result in a loss of treatment benefit as irreversible damage may have already been done.

Moreover, the practice of a conservative approach of not treating, seems to originate from uncertainty regarding the management of PDA rather than evidence favouring no intervention. This is because most studies conducted to date have involved more mature preterm babies (over 1,000 g or 28 weeks of gestation) whose PDA is more likely to close spontaneously. The studies were also largely designed to assess PDA closure rates rather than clinically important outcomes.

It is now suggested that large PDAs (those with a diameter of ≥1.5 mm) through which blood flow is pulsatile and unrestricted are less likely to close spontaneously. Targeted early treatment of large PDAs whilst asymptomatic has the potential to overcome the disadvantages of both the prophylactic and symptomatic approaches. Although clinical detection of PDA whilst asymptomatic is challenging, it can be assessed using bedside echocardiography.

Non-steroidal anti-inflammatory drugs, especially indomethacin and ibuprofen have been widely used for the treatment of PDA. Short term efficacy of indomethacin and ibuprofen are equivalent in the treatment of PDA [Su BH et al, 2008]. Ibuprofen however appears to reduce the risk of NEC and is associated with fewer clinical gastrointestinal and renal side effects compared to indomethacin; hence it is the drug of choice for this trial. Paracetamol has also been recently reported in case studies for closure of symptomatic PDA but further research needs to be done to establish its effectiveness [Oncel MY et al, 2013].

The aim of this trial is to examine whether the pharmacological closure of a large PDA (identified by echocardiography) in extremely preterm babies whilst asymptomatic has a clinically important impact on both short- and long-term health and economic outcomes.

2.2 Objectives of the trial

2.2.1 Primary objective

To determine if selective early treatment of large PDAs (confirmed by echocardiograph) in extremely preterm babies with ibuprofen within 72 hours of birth reduces the incidence of death by 36 weeks of postmenstrual age or moderate or severe bronchopulmonary dysplasia (BPD) at 36 weeks of postmenstrual age.

2.2.2 Secondary objectives

To determine if the selective treatment of confirmed large PDAs in extremely preterm babies with ibuprofen within 72 hours of birth results in:

- A reduction in the components of the primary outcome: death by 36 weeks of postmenstrual age; moderate or severe BPD at 36 weeks of postmenstrual age, severity of BPD at 36 weeks of postmenstrual age; other secondary outcomes up to discharge (see Secondary Outcomes, section 2.6.2);

- Improved health outcomes at 2 years corrected age including survival without moderate or severe neurodevelopmental disability (long-term primary objective) and survival without respiratory morbidity (long-term secondary objective).

An economic evaluation will be carried out from the perspective of the health service. It will take the form of a cost-effectiveness analysis presented in terms of cost per major outcome averted. The major outcomes are those of the primary outcome, namely death and moderate or severe BPD by 36 weeks of postmenstrual age. Additional analyses will take place on a range of secondary outcomes and on neurodevelopmental outcomes at 2 years. The incremental cost estimate for statistically significant differences in the pre-specified outcomes in primary and subgroup analyses would be computed.

2.3 Trial design

This is a multicentre, masked, randomised, placebo-controlled parallel group trial to determine if the treatment of a large PDA with ibuprofen in extremely preterm babies (23^{+0} to 28^{+6} weeks of gestation) improves short- and long-term health outcomes, and health economic outcomes.

The main trial is preceded by an internal pilot phase which has been used to assess the suitability of trial procedures and likelihood of recruitment targets being achieved.
The entire trial is anticipated to take 82 months to complete and aims to recruit a total of approximately 730 extremely preterm babies.

2.4 Eligibility

2.4.1 Inclusion criteria

Babies will be considered eligible for inclusion into the trial if they are:

- Born at 23^{+0} to 28^{+6} weeks of gestation
- Less than 72 hours old
- Confirmed by echocardiography as having a large PDA which
  - o is at least 1.5 mm in diameter (determined by gain optimised colour Doppler) **and**
  - o has unrestrictive pulsatile (left to right) flow (ratio of flow velocity in PDA Maximum (V_{max}) to Minimum (V_{min}) > 2:1) or, growing flow pattern (< 30% right to left), and **no clinical concerns of pulmonary hypertension**

In addition:

- The responsible clinician is uncertain about whether the baby might benefit from treatment to close the PDA
- Written informed consent has been obtained from the parent(s)

2.4.2 Exclusion criteria

Babies will be excluded from participation in the trial if they have:

- No realistic prospect of survival
- Severe congenital anomaly
- Clinical or echocardiography suspicion of congenital structural heart disease that contraindicates treatment with ibuprofen
- Other conditions that would contraindicate the use of ibuprofen (active bleeding especially intracranial or gastrointestinal bleeding, coagulopathy, thrombocytopenia (platelet count <50,000), renal failure, life threatening infection, pulmonary hypertension, known or suspected necrotising enterocolitis (NEC))
- Indomethacin, ibuprofen, or paracetamol administration after birth

2.5 Interventions

Ibuprofen will be supplied as a clear sterile solution at a concentration of 5 mg/ml in ampoules. Cartons containing four 2 ml single use ampoules will be provided. Each carton

will be labelled with a unique code and in compliance with the guidance given in Annexe 13 of the European Commission's guidelines for Good Manufacturing Practice.

In the main trial, an initial loading dose of 10 mg/kg (2 ml/kg) of ibuprofen will be administered, followed by two 5 mg/kg (1 ml/kg) doses at 24 and 48 hours after the initial dose. Doses are to be calculated on the birth weight of the baby. If required, the IMP can be diluted to appropriate volume with 5% glucose or 0.9% Sodium Chloride. Each dose is to be given as a short intravenous infusion over 15 minutes. All 3 doses will be given unless there are adverse effects necessitating stoppage, as referenced in the trial protocol, section 7.9.

Placebo will be supplied as a clear sterile solution of 0.9% Sodium Chloride for injection. Cartons identical to those for ibuprofen, each containing four identical single use ampoules will be provided. Volume of IMP to be withdrawn from the ampoule will be calculated following the calculations for ibuprofen dosing.

Following randomisation, first dose should be administered soon after randomisation, after 6 hours of age and within 72 hours of birth. The recommended storage will be in line with the Summary of Product Characteristics (SmPC) and once the ampoule is opened the drug must be used immediately.

2.6 Definition of primary and secondary outcomes

2.6.1 Primary outcome

The primary outcome is defined as a composite outcome of death by 36 weeks of postmenstrual age, or moderate or severe BPD at 36 weeks of postmenstrual age.

Table 1: Severity-based diagnostic criteria for BPD

Time point of assessment:	36 weeks of postmenstrual age
Therapy with oxygen > 21% and/or respiratory support for ≥ 28 days and the following:	
Mild BPD;	Baby is breathing room air
Moderate BPD;	Baby is in 22–29% oxygen, or 0.01–1.0 L/min
Severe BPD;	FiO_2 ≥ 0.3, or low flow oxygen ≥ 1.1 L/min, or the baby is receiving any respiratory support (ventilation, CPAP, or high flow oxygen therapy) to achieve saturations of ≥ 91%

The need for oxygen is subjective and hence oxygen dependency to differentiate mild from moderate BPD will be confirmed using an 'oxygen reduction test'. This is based on the threshold at which the baby is able to maintain oxygen saturations ≥ 91% whilst breathing in air or at a given minimum FiO_2. Babies unable to maintain oxygen saturations ≥ 91% in room air will be considered to be oxygen dependent and classed as moderate BPD (oxygen requirements 22–29% or 0.01–1.0L/min by low flow nasal cannula). Those babies who can be weaned to room air and stable will be categorised as having mild BPD. This test will only apply to those babies whose oxygen requirements are < 0.3, or low flow oxygen < 1.1 L/min,

and who have not received any additional respiratory support in the previous 24 hours. Babies outside of this will not be tested, but their oxygen requirements will be captured on the relevant case report form.

2.6.1.1 Oxygen reduction test

Figure 1: Oxygen reduction test flow chart

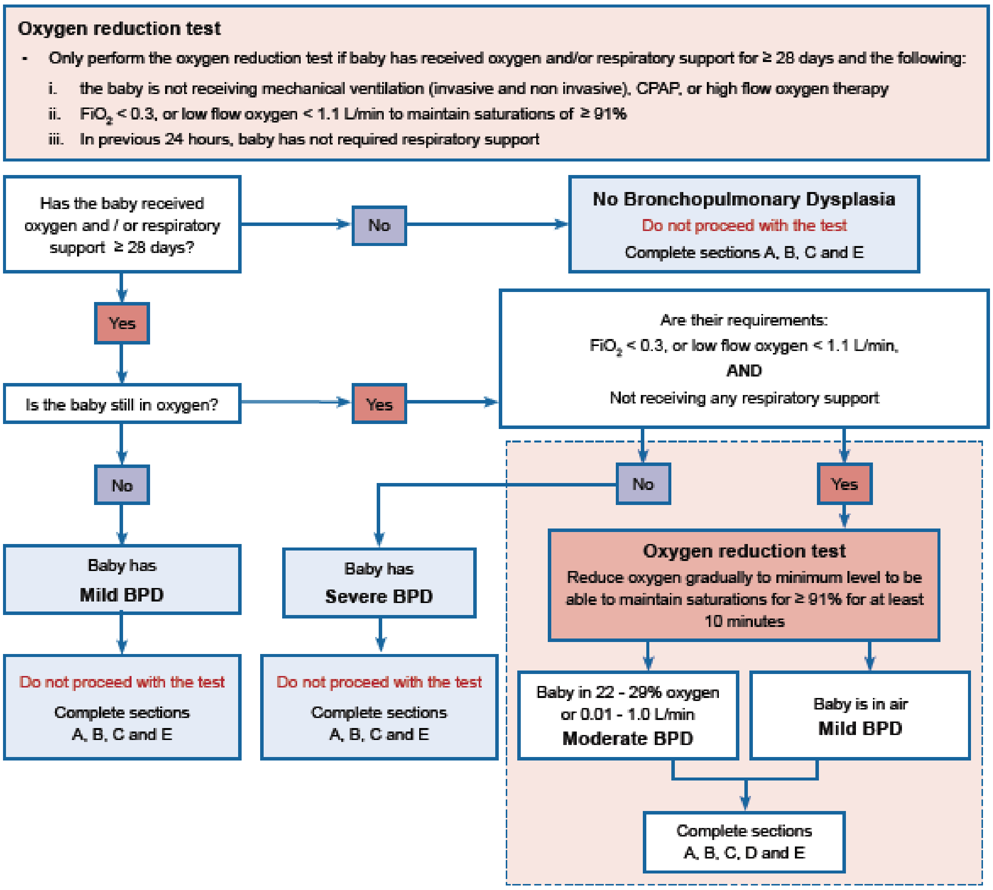

2.6.2 Secondary short-term outcomes

Due to the multiple number of short-term outcomes, and correlation between some outcomes, statistical inference will be restricted to a predefined list of tested outcomes. Summary data by trial arm will be provided for all other outcomes, but statistical tests (or the calculation of confidence intervals) will not be performed.

2.6.2.1 Secondary short-term outcomes - tested

- Death by 36 weeks' postmenstrual age
- Moderate or severe BPD at 36 weeks' postmenstrual age

Incidence or duration of the following up to discharge:

- Severe intraventricular haemorrhage (IVH) (grade III/IV with ventricular dilatation or intraparenchymal abnormality)
- Cystic periventricular leukomalacia (PVL)
- Babies treated for Retinopathy of prematurity (ROP)
- Significant pulmonary haemorrhage (fresh blood in endotracheal tube with increase in respiratory support)
- Treated for pulmonary hypertension with pulmonary vasodilator
- NEC definitive and/or complicated (Bell stage II and above) confirmed by radiology and/or histopathology
- Closed or non-significant PDA (< 1.5 mm) at around 3 weeks of age (range of 18–24 days), confirmed by ECHO (or death, or hospital discharge from recruiting centre, if discharged sooner)
- PDA ≥ 1.5 mm at around 3 weeks' (range of 18–24 days), not treated medically or by surgical closure
- Open-label treatment of a symptomatic PDA by surgical treatment
- Discharge home on oxygen
- Weight gain: a change in z score between birth and discharge (or death if sooner)

2.6.2.2 Secondary short-term outcomes - untested

- Severity of BPD at 36 weeks' postmenstrual age (see table 1 and figure 1).
- Non-cystic PVL
- Hydrocephalus
- NEC requiring surgery
- Gastrointestinal bleeding (leading to investigation or clinical treatment) within 7 days of the first dose of trial drug administration
- Spontaneous intestinal perforation
- Medical open-label treatment of a symptomatic PDA with a COX inhibitor
- Administration and duration of inotropic support
- Total duration of respiratory support
- Invasive ventilation through an endotracheal tube
- Non-invasive support through, nasal CPAP, nasal ventilation, humidified high flow nasal cannula therapy, or low flow oxygen ≥ 1.1L/min
- Duration of initial hospitalisation (birth to discharge home)
- Postnatal steroid use for chronic lung disease
- Tolerance of ibuprofen treatment within the foreseeable SAE reporting range, described in the protocol, section 9.1.4
- Head circumference: a change in head size z score between randomisation and discharge (or death if sooner).

A cost-effectiveness analysis will be conducted of deaths and BPD events avoided, which will be detailed in a separate health economics analysis plan.

2.6.3 Process outcomes

Process outcomes will measure adherence to the protocol – see section 3 for details.

2.7 Hypothesis framework

This is a superiority trial and all comparisons will be analysed and presented on this basis.

2.8 Sample size & power

Evidence from the TIPP trial suggests that the risk of death or BPD in extremely low birth weight babies at 36 weeks of postmenstrual age allocated placebo is 52% (95% CI 48% to 56%) [Schmidt et al, 2001]. However, this trial investigated the effect of prophylactic treatment and included all babies weighing 500−999g. More recent information using data derived from the latest report of Neonatal Survey Database from the Trent region (2010) provides an approximate rate of death or BPD by 36 weeks of postmenstrual age of 53% for all babies admitted to the neonatal unit. These babies would have been treated according to clinical judgement and therefore a proportion of them would have been treated with ibuprofen. Given that the risk of death or BPD in babies with a large PDA is inherently higher, it is estimated that the risk in this group is 60%.

Su et al (2008) compared ibuprofen to indomethacin in babies of ≤ 28 weeks of gestation having a PDA who were less than 24 hours old. The combined outcome of death within 30 days or BPD at 36 weeks of postmenstrual age was observed to be 42% (95% CI 29% to 55%).

It is therefore expected, given that babies will be enrolled up to 72 hours after birth, that the treatment group incidence of death/BPD at 36 weeks of postmenstrual age will be approximately 48% in the intervention arm. This would imply an absolute risk reduction of 12% (60% to 48%) in the primary outcome of the trial for babies randomised to treatment compared to placebo, which is considered a clinically important difference.

Some babies will require open-label treatment in either the treatment or placebo arm. As open-label treatment should be limited to symptomatic babies meeting only defined criteria, it is considered to have minimal or no effect on the primary outcome. Thus, adjustment of the sample size for open-label treatment is not considered necessary.

Figure 2 depicts a sample size curve for the primary outcome of the trial of death or BPD by 36 weeks of postmenstrual age, assuming 90% power, a two-sided 5% significance level and a 60% control group event rate for the primary outcome.

Figure 2: Sample size per arm vs. Difference in proportions

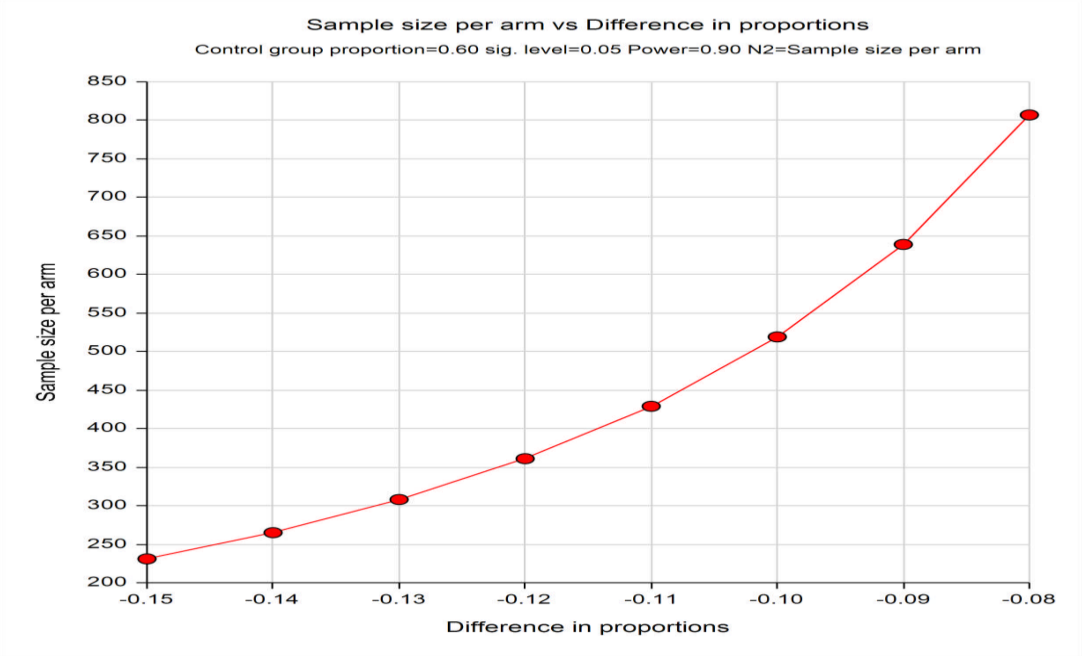

Table 2 summarises this information and allows for 1% loss to follow-up in the primary outcome. Minimal loss to follow-up is expected for the primary outcome since it is a short-term outcome and recorded whilst the baby is in hospital.

Table 2: Sample size required by event rates

Control group event rate	Active Rx group event rate	Absolute risk reduction	Relative risk reduction	Approximate total sample size required
60%	47%	13%	22%	620
**60%**	**48%**	**12%**	**20%**	**730**
60%	49%	11%	18%	870

Thus a sample size of approximately 730 babies in total (365 per arm) would be required to detect an absolute risk reduction of 12% (power 90%, 2-sided significance level of 5%) from a control group event rate of 60% to a treatment group event rate of 48%, assuming 1% lost to follow-up.

In multiple births, the babies are genetically either identical or very similar, so their outcomes are likely to be correlated. However, since multiples will be randomised independently, the loss of precision from multiples randomised to the same arm will be offset by the gain in precision from multiples randomised to the opposite arm, hence there will be a negligible impact on power.

2.9 Intervention allocation

Treatment allocation of ibuprofen or placebo will be in a ratio of 1:1 and masked such that the allocation will not be known by clinicians, the baby's family or the trial outcome assessors.

Randomisation will be managed via a secure web-based randomisation facility hosted by the NPEU CTU with telephone back-up available at all times (24/7, 365 days a year). The randomisation program will use a minimisation algorithm to ensure balance between the groups with respect to the size of the PDA (1.5 mm to < 2.0 mm; 2.0 mm to < 3.0 mm; ≥ 3.0 mm), gestational age at birth (23 to 23^{+6} weeks; 24 to 24^{+6} weeks; 25 to 25^{+6} weeks; 26 to 26^{+6} weeks; 27 to 27^{+6} weeks; 28 to 28^{+6} weeks), age at randomisation (< 12 hours; 12 to < 24 hours; 24 to < 48 hours; 48 to < 72 hours), sex (male; female or indeterminate), trial site, multiple births, mode of respiratory support at randomisation ((1) invasive ventilation (by an endotracheal tube); or (2) non-invasive respiratory support through, nasal CPAP, nasal ventilation, humidified high flow nasal cannula therapy or, low flow oxygen ≥ 1.1L/min; or (3) receiving no mechanical ventilation, or pressure support (in room air, or low flow oxygen <1.1 L/min, or ambient oxygen)), and receiving inotropes or not at the time of randomisation. Babies of multiple births will be randomised individually.

The Senior Trials Programmer at the NPEU CTU will write the randomisation program and hold the treatment allocation codes. If necessary, the code may be broken for a single baby at the request of the site PI or clinician in charge of the baby.

2.10 Data collection schedule

Data will be collected using paper case report forms (CRFs) at the centres and entered into the study's OpenClinica electronic database by NPEU CTU staff.

The CRFs to be completed are as follows:

- Form 1: Trial entry
- Form 2: Trial medication
- Form 3: ECHO results
- Form 4: 36-week form
- Form 5: Open label treatment of PDA
- Form 6: Baby outcomes
- Form 6a: Necrotising enterocolitis report form
- Form 7: Baby withdrawal form
- Form 8a: SAE report form
- Form 8b: SAE assessment form
- Form 8c: SAE processing form
- Form 9a: Incident and deviation form
- Form 9b: Incident deviation and serious breach form

Additionally, a two-year follow-up questionnaire will be sent to parents of participating infants when they reach 2 years of age. These will be completed by the parents, either on the paper questionnaire or an online form, followed by data entry into the study's OpenClinica database by NPEU CTU staff, where necessary.

Table 3 summarises the schedule of data collection and trial assessments.

Table 3: Trial assessments

Procedure	Baby Hospitalisation					
	Screening[1]	Trial Entry and Treatment (days 1–3)	Up to 7 days after trial medication	3 weeks of Age	36 weeks of PMA[8]	Discharge
Demography[7]		✓				✓
Echocardiogram/Colour Doppler[6]	✓			✓		
Confirmation of Eligibility	✓					
Consent		✓				
Randomisation[2]		✓				
Ibuprofen/Placebo Dosing[3]		✓				
IVH / PVL ultrasound scans[8]			✓		✓	
NEC						✓
Oxygen Reduction Test					✓	
SAEs[4]		✓	✓			
Concomitant Medication[5]	✓	✓		✓	✓	✓

1 Screening assessments to be completed sufficiently in advance to enable randomisation and dosing within 72 hours of birth. If consent cannot be obtained before echocardiographic evaluation for eligibility, echocardiographic assessment should continue, and consent obtained when possible if a baby is deemed eligible.

2 Randomisation to be completed sufficiently in advance to enable dosing within 72 hours of birth.

3 Initial trial drug administrations to be given soon after randomisation, after 6 hours of age and within 72 hours of birth. Subsequent doses to be administered 24 hours after the initial dose.

4 Only adverse events which are serious will be recorded from first dose until 7 days after trial medication. Only unforeseeable SAEs will be reported.

5 Concomitant medications to be recorded only in relation to unforeseeable SAEs. In the event of an unforeseeable SAE all concomitant medication, including medication given to the baby's mother, 7 days prior to the onset of the event to the time of its resolution must be recorded on the SAE form.

6 An echocardiogram scan will be performed when the baby reaches around 3 weeks of age (range of 18–24 days) or at hospital discharge if discharged earlier.

7 Demography and medications will be assessed through the PARCA-R and other questionnaires.

8 If a baby transfers from the recruiting site to a continuing care site for on-going care details of any scan would be helpful.

2.11 Interim analyses and stopping rules

A Data Monitoring Committee (DMC), independent of the applicants and of the Trial Steering Committee (TSC), will review the progress of the trial at least annually and provide advice on the conduct of the trial to the TSC and (via the TSC) to the HTA. The committee

will periodically review trial progress and outcomes as well as secondary outcomes (e.g. death, severe IVH, etc.).

Interim analyses will be supplied, in strict confidence, to the DMC, as frequently as the Chair requests. The DMC will aim to meet in person at least annually, or more often as appropriate. At the request of the DMC, interim meetings, in person or by teleconference, will be organised. Major trial issues may need to be dealt with between meetings, by phone or by email.

The DMC will be blinded to treatment allocations. The trial statistician will bring a sealed envelope containing the treatment allocations (provided by Head of Trials Programming) that can be opened to break the blind, if considered necessary.

In the light of interim data and other evidence from relevant studies, the DMC will inform the TSC if, in its view, there is proof beyond reasonable doubt that the data indicate that the trial should be terminated. A decision to inform the TSC of such a finding will in part be based on statistical considerations. Appropriate proof beyond reasonable doubt cannot be specified precisely. A difference of at least 3 standard errors in the interim analysis of a major endpoint may be needed to justify halting or modifying the study prematurely.

2.12 Trial reporting

The trial will be reported according to the principles of the CONSORT statement.

3 Protocol non-compliances

A protocol non-compliance is defined as a failure to adhere to the protocol such as the wrong intervention being administered, incorrect data being collected and documented, errors in applying inclusion/exclusion criteria or missed follow-up visits due to error.

All protocol non-compliances will be listed in the final report. Non-compliances are defined below.

3.1 Major

The following are pre-defined major protocol non-compliances with a direct bearing on the primary outcome:

- Data considered to be fraudulent

3.2 Minor

The following will be defined as minor protocol non-compliances:

Participants randomised in error

These include infants:

- who are < 23 weeks or ≥ 29 weeks of gestation
- who are ≥ 72 hours old
- with a PDA < 1.5 mm in diameter OR who does not have unrestrictive pulsatile left to right flow or, growing pattern with right to left flow of 30% or more

- who have clinical or echocardiography evidence of pulmonary hypertension
- where written informed consent has not been obtained from the parent(s)
- with a severe congenital anomaly
- with contraindications to the use of ibuprofen
- who have had indomethacin, ibuprofen, or paracetamol administered after birth.

Treatment non-compliances
These include infants who:

- do not receive allocated intervention. These include infants who were allocated ibuprofen, who instead received placebo, and vice versa.
- do not receive the correct number of doses. These include infants who received less than 3 doses of the trial medication.
- do not receive medication at the correct time. These include infants who received their first dose later than 72 hours after birth, or received their 2nd or 3rd dose outside the specified dosing window (< 18 hours or > 72 hours between doses 1 and 2, or doses 2 and 3; or dose 3 completed > 7 days after first dose administered).
- received open-label treatment without meeting the criteria. These include infants who received open-label treatment but did not meet the defined criteria for doing so:

  1. Inability to wean on ventilator (ventilated for at least 7 days continuously) and any of: inability to wean oxygen; persistent hypotension; pulmonary haemorrhage; signs of cardiac failure,
  AND
  2. Echocardiographic findings of a large PDA (PDA ≥ 2.0 mm with pulsatile flow)
  AND
  3. Echocardiographic findings of hyperdynamic circulation or ductal steal.

Trial procedure non-compliances:

- ECHO not done around 3 weeks (18–24 days) of age
- Oxygen reduction test not done when baby was eligible

Protocol non-compliance will be reported in a process outcomes table.

4 Analysis populations

4.1 Post-randomisation exclusions

Exclusions to the analysis post-randomisation are defined as any of the following:

- Infants for whom a written consent form from the parent(s) was not received
- Infants for whom consent to use their data was withdrawn by the parent(s)

- Infants for whom an entire record of fraudulent data was detected (should fraudulent data be detected, consideration will be given to excluding all data for the site where such data were found).

4.2 Population definitions

4.2.1 Intention to treat population

The intention to treat (ITT) population will be all infants randomised, excluding post-randomisation exclusions.

4.2.2 Interim analysis population

Different denominators will be used in the interim analysis:
- Baseline data will be reported for all trial participants with available data, excluding known post-randomisation exclusions.
- Outcome data will be reported for babies who can be described as 'completers', i.e. all trial participants with available data who have died or been discharged home, excluding post-randomisation exclusions.
- Process outcomes will be reported for all trial participants with available data, excluding known post-randomisation exclusions.
- Safety data will be reported for all trial participants who received at least one dose of the study drug.

4.2.3 Safety population

All infants randomised who received at least one dose of the study drug.

5 Descriptive analyses

5.1 Representativeness of trial population and participant throughput

The flow of participants through each stage of the trial will be summarised by randomised group using a figure presenting the flow of participants. This will describe the numbers of infants:

- Assessed for eligibility
- Eligible
- Randomised
- Allocated to ibuprofen
    - Did not receive allocated treatment (with reasons)
    - Randomised in error
- Allocated to placebo
    - Did not receive allocated treatment (with reasons)

    - Randomised in error
- Withdrawals
- Included in safety population
- Post-randomisation exclusions (with reasons)
- Included in the ITT population

5.2 Baseline comparability of randomised groups

Baseline demographic and clinical characteristics at trial entry will be described for all infants and their mothers in the ITT population by randomised group. The following characteristics will be described:

Mother's baseline characteristics:

- Ethnicity
- Age (years)
- Deprivation index
- Antenatal steroid use (any)
    - < 24 hours before birth
    - ≥ 24 hours before birth
- Antenatal COX inhibitor use
- Antenatal magnesium sulphate use for neuroprotection

Infant's characteristics at trial entry

- Enrolling centre
- Born in enrolling centre
- Postnatal age at randomisation (hours)
- Gestational age at birth (weeks)
- Mode of delivery
- Forceps or Ventouse used in delivery
- Main cause of preterm birth
- Birth weight (g)
- Birth weight z score
- Head circumference (cm)
- Head circumference z score
- Sex
- Baby is one of a multiple pregnancy
- Sibling enrolled in the study (in multiple pregnancies)
- APGAR score 5 minutes after birth
- Baby's worst base excess at first hour after birth
- CRIB II (without temperature) [Parry G et al, 2003]
- Size of PDA
- Mode of respiratory support at randomisation
- Receiving inotropes at randomisation

The number and percentage will be presented for binary and categorical variables. The mean and standard deviation or the median and the interquartile range will be presented for continuous variables, and the range if appropriate. There will be no tests of statistical

significance performed nor confidence intervals calculated for differences between randomised groups on any baseline variable.

5.3 Losses to follow-up

Minimal loss to follow-up is expected for the primary outcome since it is a short-term outcome and recorded whilst the baby is in hospital.

6 Comparative analyses

Infants will be analysed according to their allocation, regardless of the intervention they received (ITT population). The placebo group will be used as the reference group in all analyses.

Outcomes will be summarised with counts and percentages for categorical variables, means and standard deviations for normally distributed continuous variables, or median and interquartile range for other non-normally distributed continuous or time-to-event variables.

For binary outcomes, risk ratios and confidence intervals will be calculated using log binomial regression, and if a model fails to converge a Poisson regression model with a robust variance estimator will be used. Continuous outcomes will be analysed using linear regression models, with mean differences and confidence intervals presented for approximately normally distributed outcomes. Skewed continuous outcomes will be analysed using quantile regression models, with median differences and confidence intervals presented. Time-to-event outcomes will be analysed using Cox regression and hazard ratios with confidence intervals will be presented.

Analyses will be adjusted for all minimisation factors where possible. Centre will be treated as a random effect in the model, and all other factors as fixed effects, including multiple births. Correlation between siblings from multiple births will be accounted for in the adjusted model by nesting 'multiple' cluster as a random effect within centre. All factors will be fitted as fixed effects in quantile regression, as random effects cannot be modelled using these methods of analysis. Both crude and adjusted effect estimates will be presented, but the primary inference will be based on the adjusted estimates.

Analysis of secondary outcomes will be clearly delineated from the primary outcomes in any statistical reports produced.

6.1 Detailed definition of outcomes

See appendix A for a detailed derivation for each level of BPD. Detailed derivations for all other outcomes are described in a separate document.

6.2 Primary analysis

The primary analysis for the primary outcome and all secondary outcomes will be conducted on the ITT population, adjusted for minimisation factors where possible, as described above.

6.3 Secondary analyses
A restricted analysis on the primary outcome and its components, excluding infants who received open label treatment without meeting the specified criteria.

6.4 Pre-specified subgroup analyses
Pre-specified subgroup analyses will use the statistical test of interaction and where appropriate, results will be presented as risk ratios with confidence intervals.

Pre-specified subgroups on the primary outcome and its components will be based on:
- gestational age at birth (23 to 23^{+6} weeks; 24 to 24^{+6} weeks; 25 to 25^{+6} weeks; 26 to 26^{+6} weeks; 27 to 27^{+6} weeks; 28 to 28^{+6} weeks)
- size of the PDA (1.5 mm to < 2.0 mm; 2.0 mm to < 3.0 mm; ≥ 3.0 mm)
- mode of respiratory support at randomisation (invasive ventilation (by an endotracheal tube); non-invasive respiratory support through nasal CPAP, nasal ventilation, humidified high flow nasal cannula therapy or, low flow oxygen ≥ 1.1 L/min; or receiving no mechanical ventilation, or pressure support (in room air, or low flow oxygen < 1.1 L/min, or ambient oxygen)).

A further pre-specified subgroup analysis on NEC Bell stage II and above will be conducted by size of the PDA.

6.5 Sensitivity analyses
No sensitivity analyses have been specified.

6.6 Significance levels and adjustment of p-values for multiplicity
95% confidence intervals will be used for all pre-specified outcome comparisons including subgroup analysis. Due to the large number of secondary outcomes, a pre-specified list of tested and untested outcomes has been provided (see section 2.6.2).

6.7 Missing data
Missing data will be described, for example, by presenting the number of individuals in the missing category. Missing data as a result of babies being lost to follow-up is expected to be minimal for short term outcomes.

6.8 Statistical software employed
The statistical software Stata/SE will be used for all analyses.

7 Safety data analysis

Serious adverse events (SAEs) will be listed by allocation.

In addition, the following foreseeable (SAEs) occurring within seven days after trial medication is completed will be reported by trial arm:

- Anaemia requiring transfusion

- Clinically significant intracranial abnormality on cranial ultrasound scan – intracranial haemorrhage or white matter injury
- Coagulopathy requiring treatment
- Culture proven sepsis
- Death (unless unforeseeable in this population)
- Fluid retention
- Gastrointestinal bleeding
- Haematuria
- Haemothorax
- High blood creatinine level (defined as > 100 µmol/L)
- Hyperbilirubinemia necessitating exchange transfusion
- Hyperglycaemia
- Hypoglycaemia
- Hypotension treated with inotropes
- Impaired renal function (urine output < 0.5 ml/kg/hour, and or serum creatinine > 100 µmol/L)
- Low serum sodium level/hyponatremia (defined as sodium < 130 mmol/L)
- Necrotising enterocolitis
- Neutropenia (defined as < 1.0 mmol/L)
- Pneumothorax requiring treatment
- Pulmonary hypertension requiring treatment with pulmonary vasodilator
- Respiratory failure
- Seizures requiring treatment
- Significant pulmonary haemorrhage
- Spontaneous intestinal perforation
- Thrombocytopenia

8 Additional exploratory analysis

Any analyses not specified in the analysis protocol will be exploratory in nature and will be documented in a separate statistical analysis plan. Any post-hoc analysis requested by the oversight committees, a journal editor or referees will be labelled explicitly as such.

9 Deviation from analysis described in protocol

None yet.

10 Appendix A – Detailed derivation of levels of BPD

__Rules:__

- If oxygen reduction test results are too early or too late (e.g. outside ±1 week), oxygen requirements nearer 36 weeks will be requested, to be added to a new CRF if possible.

- If oxygen reduction test was not done, but oxygen requirements were not provided – accept severity given in form 4, C3 (mild or severe) for analysis, but use oxygen requirements where available to query if necessary.

- If baby died before 36 weeks, they will be excluded from the denominator for level of BPD.

Questions from Form 4: 36 week form

__NO BPD__

C1. Please confirm the highest level of respiratory support at 36 weeks of PMA = Receiving no mechanical support

C2. Has the baby been in oxygen > 21% and/or received respiratory support? = No

OR

Total number of days on any respiratory support: < 28

[Optional] C3. Was an oxygen reduction test carried out at 36 weeks of PMA? = No

[Optional] If no, please state the main reason why the test was not performed:

- Other (i.e. cannot be Baby has Mild or Severe BPD)

OR

[Optional] If no: Baby not eligible *= Yes [Pilot CRF]*

OR

Baby discharged before they reach 36 weeks' PMA, and 36 week form missing

__MILD__

Oxygen reduction test carried out:

C1. Please confirm the highest level of respiratory support at 36 weeks of PMA = Receiving no mechanical support

C2. Has the baby been in oxygen > 21% and/or received respiratory support? = Yes

Total number of days of any respiratory support: ≥ 28

C3. Was an oxygen reduction test carried out at 36 weeks of PMA? = Yes

[Check: D1. What was the date of the test? = 36 weeks' PMA OR date of discharge]

D2. What was the amount of oxygen before starting test? < 30% OR < 1.1 L/min

D3. Was the baby able to maintain saturations (in air) ≥ 91% for 10 minutes? = Yes

OR

Oxygen reduction test not carried out:

C1. Please confirm the highest level of respiratory support at 36 weeks of PMA = Receiving no mechanical ventilation or pressure support

C2. Has the baby been in oxygen > 21% and/or received respiratory support? = Yes

Total number of days of any respiratory support: ≥ 28

C3. Was an oxygen reduction test carried out at 36 weeks of PMA? = No

If no, please state the main reason why the test was not performed:

Baby has Mild BPD = Yes

AND (OPTIONALLY)

Oxygen requirement at 36 weeks PMA = Missing

OR Receiving no mechanical support and in room air [Pilot CRF]

OR

Discharged before 36 weeks' PMA:

Infant was discharged before 36 weeks' PMA AND:

C1. Please confirm the highest level of respiratory support at 36 weeks of PMA = Receiving no mechanical ventilation or pressure support

C2. Has the baby been in oxygen > 21% and/or received respiratory support? = Yes

Total number of days of any respiratory support: ≥ 28

<u>**MODERATE**</u>

Oxygen test was carried out:

C1. Please confirm the highest level of respiratory support at 36 weeks of PMA = Receiving no mechanical ventilation or pressure support

C2. Has the baby been in oxygen > 21% and/or received respiratory support? = Yes

Total number of days of any respiratory support: ≥ 28

C3. Was an oxygen reduction test carried out at 36 weeks of PMA? = Yes

[Check: D1. What was the date of the test? = 36 weeks' PMA OR date of discharge]

D2. What was the amount of oxygen before starting test? < 30% OR < 1.1 L/min

D3. Was the baby able to maintain saturations (in air) ≥ 91% for 10 minutes? = No

If no, how much oxygen did the baby require to maintain saturations for ≥ 91%? 22-29% or 0.01–1.0 L/min

AND (OPTIONALLY)

D4. Was the test stopped prematurely due to clinical deterioration? = Yes

OR

Oxygen test was not carried out when it should have been:

C1. Please confirm the highest level of respiratory support at 36 weeks of PMA = Receiving no mechanical ventilation or pressure support
C2. Has the baby been in oxygen > 21% and/or received respiratory support? = Yes
Total number of days of any respiratory support: ≥ 28
C3. Was an oxygen reduction test carried out at 36 weeks of PMA? = No
If no, please state the main reason why the test was not performed:

- Other (i.e. cannot be Baby has Mild or Severe BPD)

OR Baby not eligible (as per flow diagram) = No [Pilot CRF]
If oxygen reduction test not done in an otherwise eligible baby:
Oxygen requirement at 36 weeks of PMA: < 30% OR < 1.1 L/min

<u>**SEVERE**</u>
C1. Please confirm the highest level of respiratory support at 36 weeks of PMA = Invasive ventilation OR Non-invasive respiratory support
OR
C2. Has the baby been in oxygen > 21% and/or received respiratory support? = Yes
Total number of days of any respiratory support: ≥ 28
C3. Was an oxygen reduction test carried out at 36 weeks of PMA? = No
If no, please state the main reason why the test was not performed = Baby has Severe BPD
OR Baby not eligible = Yes [Pilot CRF]
AND (OPTIONALLY)
Oxygen requirement at 36 weeks of PMA ≥ 30% OR ≥ 1.1 L/min

11 References

11.1 Trial documents

- Baby-OSCAR Dummy tables
- Baby-OSCAR Data derivation document

11.2 Other references

Gamble C, Krishan A, Stocken D, Lewis S, Juszczak E, Doré C, Williamson PR, Altman DG, Montgomery A, Lim P, Berlin J, Senn S, Day S, Barbachano Y, Loder E. Guidelines for the Content of Statistical Analysis Plans in Clinical Trials. *JAMA* 2017;318(23):2337–43.

Neonatal Survey Database from the Trent Region 2010. Available from: http://www.le.ac.uk/departments/health-sciences/research/timms/projects/tns

Noori S, McCoy M, Friedlich P, Bright B, Gottipati V, Seri I, Sekar K. Failure of ductus arteriosus closure is associated with increased mortality in preterm infants. *Pediatrics* 2009;123; 138–e144.

Oncel MY, Yurttutan S, Degirmencioglu H, Uras N, Altug N, Erdeve O, Dilmen U: Intravenous paracetamol treatment in the management of patent ductus arteriosus in extremely low birth weight infants. *Neonatology* 2013;103:166−9.

Oncel MY, Yurttutan S, Uras N, Altug N, Ozdemir R, Ekmen S, Erdeve O, Dilmen U: An alternative drug (paracetamol) in the management of patent ductus arteriosus in ibuprofen- resistant or contraindicated preterm infants. *Arch Dis Child Fetal Neonatal Ed* 2013;98:94.

Parry G, Tucker J, Tarnow-Mordi W, UK Neonatal Staffing Study Collaborative Group. CRIB II: an update of the clinical risk index for babies score. *Lancet* 2003 May 24;361(9371):1789-91

Schmidt B, Davis P, Moddemann D, Ohlsson A, Roberts RS, Saigal S, et al. Long-term effects of indomethacin prophylaxis in extremely-low-birth-weight infants. *N Engl J Med* 2001;344(26):1966−72.

Su BH, Lin HC, Chiu HY, Hsieh HY, Chen HH, Tsai YC. Comparison of ibuprofen and indometacin for early-targeted treatment of patent ductus arteriosus in extremely premature infants: a randomised controlled trial. *Arch Dis Child Fetal Neonatal Ed* 2008;93(2):F94−9.

12 Approval

Lead Medical Statistician	Name: Associate Professor Louise Linsell	
	Signature llinsell<br>llinsell (Feb 10, 2021 17:07 GMT)	Date<br>Feb 10, 2021
Chief Investigator	Name: Professor Samir Gupta	
	Signature<br>Samir Gupta (Feb 13, 2021 10:10 GMT+3)	Date<br>Feb 13, 2021
Chair of Trial Steering Committee (or delegate)	Name: Emeritus Professor Michael Weindling	
	Signature AMWeindling<br>AMWeindling (Feb 13, 2021 18:47 GMT)	Date<br>Feb 13, 2021

Document history

Version	Date	Edited by	Comments/Justification	Timing in relation to interim analysis/ unblinding
0.1	22/07/2015	JB	For review by PH	Not yet unblinded
0.2	04/08/2015	JB	Revised following comments from PH	Not yet unblinded
0.3	21/09/2017	JB	Comments from SG received. Transferred to new NPEU CTU SAP template.	Not yet unblinded
0.4	22/09/2017	JB	Revised following comments from LL. Added list of abbreviations.	Not yet unblinded
0.5	26/09/2017	JB	Revised following review by SG	Not yet unblinded
0.6	11/10/2017	JB	Revised following review by LL	Not yet unblinded
0.7	16/07/2018	JB	Revised following review by TSC	Not yet unblinded
0.8	04/02/2020	JB	Updated following discussion with collaborators, discussion with LL, and comparison with protocol amendment	Not yet unblinded
0.9	08/12/2020	LL	Edits prior to sign off	Not yet unblinded
0.91	22/12/2020	EJ	Edits prior to sign off – grammar, typos, inconsistencies	Not yet unblinded
0.92	23/12/2020	EJ	Further edits	Not yet unblinded
0.93	06/01/2021	LL	Edits accepted and comments tidied	Not yet unblinded
1.0	08/02/2021	JB	Sensitivity analyses removed with agreement from LL.<br>Final version.	Not yet unblinded

Baby-OSCAR SAP v1.0 21_02_08

Final Audit Report 2021-02-13

Created:	2021-02-10
By:	Ann Kennedy (ann.kennedy@npeu.ox.ac.uk)
Status:	Signed
Transaction ID:	CBJCHBCAABAAuZAafUeShuUOUnnvZOWYRv39Iks2Wu_6

"Baby-OSCAR SAP v1.0 21_02_08" History

Document created by Ann Kennedy (ann.kennedy@npeu.ox.ac.uk)
2021-02-10 - 4:54:55 PM GMT- IP address: 163.1.206.129

Document emailed to llinsell (louise.linsell@npeu.ox.ac.uk) for signature
2021-02-10 - 4:57:55 PM GMT

Email viewed by llinsell (louise.linsell@npeu.ox.ac.uk)
2021-02-10 - 5:07:08 PM GMT- IP address: 163.1.206.129

Document e-signed by llinsell (louise.linsell@npeu.ox.ac.uk)
Signature Date: 2021-02-10 - 5:07:24 PM GMT - Time Source: server- IP address: 163.1.206.129

Document emailed to Samir Gupta (samir.gupta@durham.ac.uk) for signature
2021-02-10 - 5:07:26 PM GMT

Email viewed by Samir Gupta (samir.gupta@durham.ac.uk)
2021-02-13 - 7:09:28 AM GMT- IP address: 212.70.107.74

Document e-signed by Samir Gupta (samir.gupta@durham.ac.uk)
Signature Date: 2021-02-13 - 7:10:02 AM GMT - Time Source: server- IP address: 212.70.107.74

Document emailed to AMWeindling (a.m.weindling@liverpool.ac.uk) for signature
2021-02-13 - 7:10:04 AM GMT

Email viewed by AMWeindling (a.m.weindling@liverpool.ac.uk)
2021-02-13 - 6:14:34 PM GMT- IP address: 86.164.161.176

Document e-signed by AMWeindling (a.m.weindling@liverpool.ac.uk)
Signature Date: 2021-02-13 - 6:47:18 PM GMT - Time Source: server- IP address: 86.164.161.176

Agreement completed.
2021-02-13 - 6:47:18 PM GMT

Adobe Sign
